# The dynamics of human bone marrow adipose tissue in response to feeding and fasting

**DOI:** 10.1172/jci.insight.138636

**Published:** 2021-06-22

**Authors:** Pouneh K. Fazeli, Miriam A. Bredella, Gisela Pachon-Peña, Wenxiu Zhao, Xun Zhang, Alexander T. Faje, Megi Resulaj, Sai P. Polineni, Tara M. Holmes, Hang Lee, Elizabeth K. O’Donnell, Ormond A. MacDougald, Mark C. Horowitz, Clifford J. Rosen, Anne Klibanski

**Affiliations:** 1 Neuroendocrine Unit, Massachusetts General Hospital, Boston, Massachusetts, USA.; 2 Harvard Medical School, Boston, Massachusetts, USA.; 3 Neuroendocrinology Unit, Division of Endocrinology and Metabolism, Department of Medicine, School of Medicine, University of Pittsburgh, Pittsburgh, Pennsylvania, USA.; 4 Department of Radiology, Massachusetts General Hospital, Boston, Massachusetts, USA.; 5 Maine Medical Center Research Institute, Scarborough, Maine, USA.; 6 Translational and Clinical Research Center,; 7 Biostatistics Center, and; 8 Division of Hematology/Oncology, Massachusetts General Hospital, Boston, Massachusetts, USA.; 9 Department of Molecular & Integrative Physiology, University of Michigan Medical School, Ann Arbor, Michigan, USA.; 10 Department of Orthopaedics and Rehabilitation, Yale School of Medicine, New Haven, Connecticut, USA.

**Keywords:** Metabolism, Adipose tissue, Bone marrow

## Abstract

**BACKGROUND:**

Adipocytes were long considered inert components of the bone marrow niche, but mouse and human models suggest bone marrow adipose tissue (BMAT) is dynamic and responsive to hormonal and nutrient cues.

**METHODS:**

In this study of healthy volunteers, we investigated how BMAT responds to acute nutrient changes, including analyses of endocrine determinants and paracrine factors from marrow aspirates. Study participants underwent a 10-day high-calorie protocol, followed by a 10-day fast.

**RESULTS:**

We demonstrate (a) vertebral BMAT increased significantly during high-calorie feeding and fasting, suggesting BMAT may have different functions in states of caloric excess compared with caloric deprivation; (b) ghrelin, which decreased in response to high-calorie feeding and fasting, was inversely associated with changes in BMAT; and (c) in response to high-calorie feeding, resistin levels in the marrow sera, but not the circulation, rose significantly. In addition, TNF-α expression in marrow adipocytes increased with high-calorie feeding and decreased upon fasting.

**CONCLUSION:**

High-calorie feeding, but not fasting, induces an immune response in bone marrow similar to what has been reported in peripheral adipose tissue. Understanding the immunomodulatory regulators in the marrow may provide further insight into the homeostatic function of this unique adipose tissue depot.

**FUNDING:**

NIH grant R24 DK084970, Harvard Catalyst/The Harvard Clinical and Translational Science Center (National Center for Advancing Translational Sciences, NIH, award UL 1TR002541), and NIH grants P30 DK040561 and U19 AG060917S1.

## Introduction

Bone marrow adipose tissue (BMAT) is a major component of the bone marrow microenvironment, yet its function is not well understood. Although individuals with anorexia nervosa, a psychiatric illness characterized by self-induced starvation and low body weight and a model of chronic starvation, have low levels of subcutaneous (SAT) and visceral adipose tissue (VAT) (1), BMAT levels are higher in girls and women with anorexia nervosa compared with normal-weight controls (2, 3). Why this adipose depot would be expanded during chronic starvation while other lipid depots are depleted is unknown, but the paradoxical behavior of this lipid depot may provide insight into its function.

The rapidity of energy changes appears to affect BMAT changes. We previously demonstrated that women with a history of anorexia nervosa who are now at normal body weight have lower levels of BMAT compared with low-weight women with active anorexia nervosa (4). In contrast, with more rapid weight changes (≤12 months), BMAT increases with weight gain and decreases with weight loss, suggesting that this adipose tissue depot behaves differently with subacute fluctuations in nutrient status and is a dynamic compartment (5). This observation is consistent with murine models in which a high-fat diet increases BMAT (6) and surgically induced weight loss leads to BMAT loss (7). In human models, in the setting of acute weight loss due to surgical intervention, BMAT changes have been variable depending on the type of surgery. For example, BMAT has been shown to decrease (8, 9) 6–12 months after Roux-en-Y gastric bypass, a procedure causing weight loss both through restricting food intake and malabsorption, whereas in individuals undergoing sleeve gastrectomy, a primarily restrictive procedure, BMAT in the spine and hip increases 12 months after surgery, in the setting of weight loss (10). Therefore, few data are available examining BMAT in humans in the setting of structured acute changes in weight and nutrient flux.

We hypothesized that acute nutrient changes would cause rapid changes in BMAT and that these changes would be associated with hormones and immune modulators associated with increased caloric intake, starvation, and/or body weight. We therefore investigated BMAT changes in humans in response to a structured, acute, short-term weight gain protocol followed by an acute, short-term weight loss protocol. We measured BMAT changes in response to these acute changes in body weight and nutritional status and investigated the hormonal and inflammatory predictors of these changes.

## Results

### Baseline characteristics of study population

Twenty-three subjects (*n* = 10 women) were admitted to the Translational and Clinical Research Center (TCRC) at the Massachusetts General Hospital for a 10-day inpatient high-calorie visit. The goal of this portion of the study was to achieve a 7% weight gain during the 10-day admission. Subjects were subsequently discharged for approximately 2 weeks, during which time they were instructed to resume their normal diet, and then readmitted to the TCRC for a 7- to 10-day inpatient fast, during which time subjects were not allowed any caloric intake. Baseline characteristics of the study participants are listed in Table 1. On the first and last day of the high-calorie intake and fasting visits, we measured BMAT using ^1^H-magnetic resonance spectroscopy (^1^H-MRS) with a primary endpoint of change in BMAT at the L4 vertebra, femoral diaphysis and femoral metaphysis based on our prior work demonstrating differences in BMAT at these 2 sites in women with anorexia nervosa compared to normal-weight controls (2) and changes in BMAT at the femur with subacute changes in weight (5). We also measured hormones and inflammatory mediators that have been associated with BMI, nutrient flux — including high-calorie feeding and starvation — or those that have been shown to be determinants of BMAT in animal models (Table 1). BMAT at the L4 vertebra (*P* = 0.02), femoral diaphysis (*P* = 0.002), and femoral metaphysis (*P* = 0.004) was significantly greater in men at baseline as compared with women.

### Baseline associations between BMAT, body composition, and hormonal parameters

#### At baseline, there were significant associations between body composition parameters and BMAT as well as hormonal parameters and BMAT (Supplemental Tables 1–6; supplemental material available online with this article; https://doi.org/10.1172/jci.insight.138636DS1).

Consistent with prior studies including obese, premenopausal women (11) but in contrast to studies including only normal- and low-weight, premenopausal women (5), there were significant positive associations between VAT and BMAT at the L4 vertebra (rho = 0.57, *P* = 0.004) and the femoral metaphysis (rho = 0.59, *P* = 0.004). There was also a significant inverse association between BMAT at the femoral diaphysis and SAT (*R* = –0.49, *P* = 0.02; Supplemental Table 1). Consistent with our prior data in normal-weight and low-weight premenopausal women (5), there was a significant inverse association between leptin and BMAT at the femoral diaphysis at baseline (rho = –0.43, *P* = 0.04); in contrast to these prior data, there was an inverse association between adiponectin and BMAT at the femoral metaphysis (rho = –0.42, *P* = 0.049; ref. 5 and Supplemental Table 2).

In the women, similar to the group as a whole, there was a significant positive association between VAT and BMAT at the L4 vertebra (*R* = 0.73, *P* = 0.02) and femoral metaphysis (rho = 0.64, *P* = 0.048). In female subjects, there was also a positive association between SAT and BMAT at the L4 vertebra (*R* = 0.66, *P* = 0.04), in contrast to what we previously observed in a population of normal-weight and low-weight, premenopausal women (ref. 5 and Supplemental Table 3). In women, in contrast to animal models in which FGF21-transgenic mice have higher bone marrow adipocyte number and area (12), there was an inverse association between FGF21 and BMAT at the L4 vertebra (*R* = –0.64, *P* = 0.048; Supplemental Table 4).

In the men, there were no significant associations between VAT and BMAT, but there was an inverse association between BMAT at the femoral diaphysis and BMI (rho = –0.57, *P* = 0.04; Supplemental Table 5). In men, there was a significant positive association between ghrelin and BMAT at the femoral diaphysis (*R* = 0.56, *P* = 0.045), consistent with data in a rodent model demonstrating increased bone marrow adiposity in response to infusion with ghrelin (ref. 13 and Supplemental Table 6).

### Changes in weight, body composition parameters, and BMAT with high-calorie feeding, with fasting, and during the 2-week stabilization period

#### High-calorie visit.

**Figure 1:**
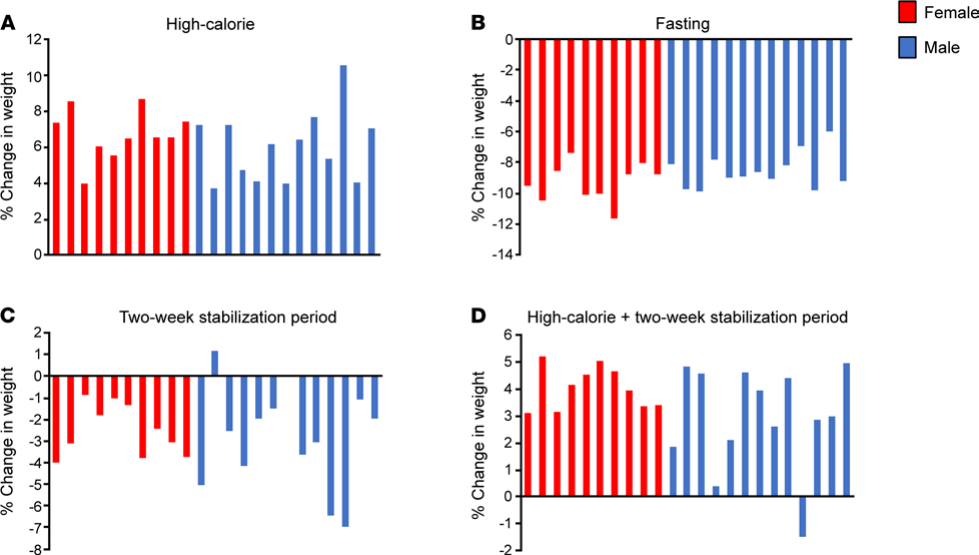
Weight changes in individual subjects during the study. Weight changes during high-calorie feeding (**A**), fasting (**B**), and 2-week stabilization period (**C**) as well as percentage change from baseline weight at the conclusion of the 2-week stabilization period (**D**). Each bar represents an individual subject. Percentage change in weight ranged from 3.6% to 10.5% during the high-calorie visit (**A**). During the fasting visit, percentage change in weight ranged from –5.9% to –11.6% (**B**). Percentage change in weight ranged from –6.9% to 1.1% during the 2-week stabilization period (**C**). One male subject was at a lower weight (–1.5%) as compared with his baseline weight at the conclusion of the 2-week stabilization period, but all other subjects were at a higher weight compared with baseline, ranging from 0.4% to 5.1% higher than baseline weight at the conclusion of the 2-week stabilization period (**D**).

Body weight increased a mean of 6.3% ± 0.4% during the high-calorie visit (*P* < 0.0001; Table 2 and Figure 1A). SAT (mean increase of 13.6% ± 2.5%, *P* < 0.0001), VAT (mean increase: 28.1% ± 8.3%, *P* = 0.006), trunk fat (mean increase: 9.1% ± 1.2%, *P* < 0.0001), extremity fat (mean increase: 7.6% ± 0.9%, *P* < 0.0001), and lean mass (mean increase: 5.6% ± 0.6%, *P* < 0.0001) all increased during the high-calorie visit (Table 2), although changes in lean mass measured by DXA may be attributable to shifts in total body water (Table 2).

**Figure 2:**
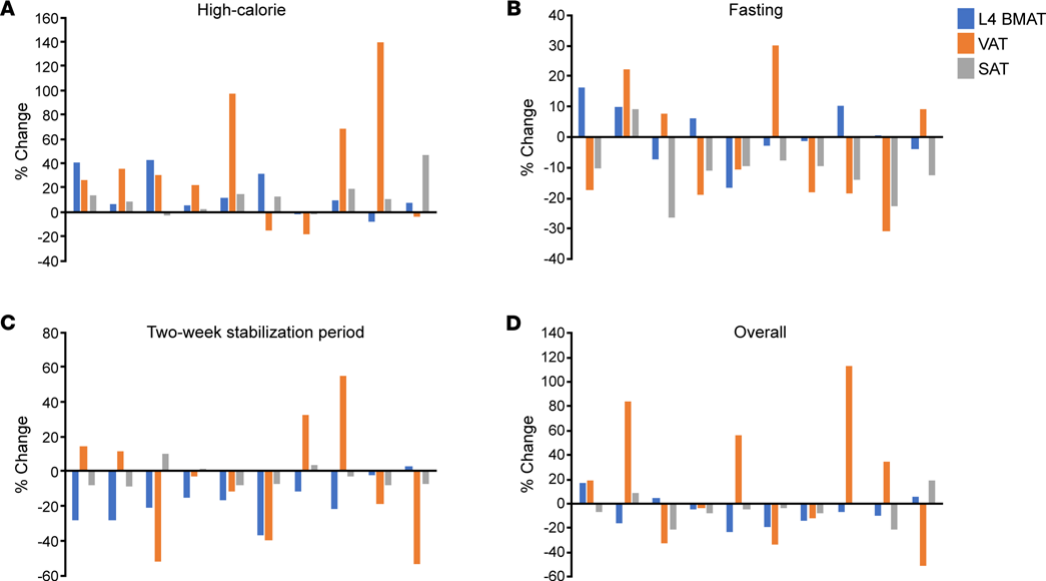
Changes in L4 vertebral BMAT, VAT, and SAT during the study in women. Changes in L4 vertebral BMAT, VAT, and SAT during high-calorie feeding (**A**), during fasting (**B**), during 2-week stabilization period (**C**), and overall (**D**). Each cluster of 3 bars represents an individual female subject, and subjects are listed in ascending order based on baseline BMI (the first cluster in each graph represents a female subject with a baseline BMI of 23.3 kg/m^2^; the last cluster in each graph represents a female subject with a baseline BMI of 27.9 kg/m^2^). During high-calorie feeding, L4 vertebral BMAT increased in 80% of women, VAT increased in 70% of women, and SAT increased in 80% of women (**A**). During fasting, L4 vertebral BMAT increased in 50% of women, VAT decreased in 60% of women, and SAT decreased in 90% of women (**B**). During the 2-week stabilization period, L4 vertebral BMAT decreased in 90% of women, VAT decreased in 60% of women, and SAT decreased in 70% of women (**C**). Compared with baseline, at the end of the study, L4 vertebral BMAT decreased in 70% of women, VAT decreased in 50% of women, and SAT decreased in 80% of women (**D**).

**Figure 3:**
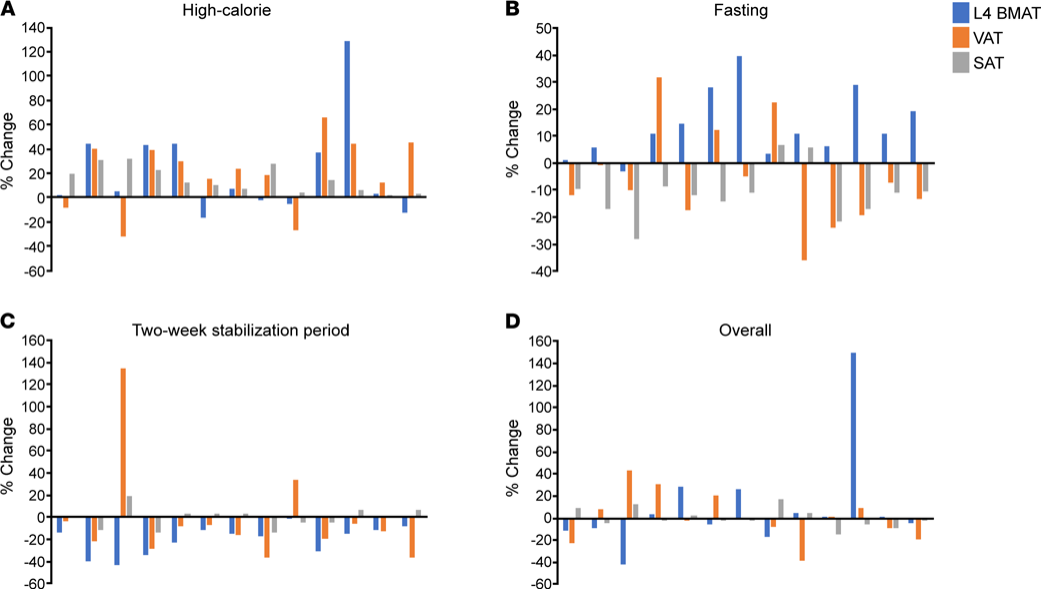
Changes in L4 vertebral BMAT, VAT, and SAT during the study in men. Changes in L4 vertebral BMAT, VAT, and SAT during high-calorie feeding (**A**), during fasting (**B**), during 2-week stabilization period (**C**), and overall (**D**). Each cluster of 3 bars represents an individual male subject; subjects are listed in ascending order based on baseline BMI (the first cluster in each graph represents a male subject with a baseline BMI of 23.4 kg/m^2^; the last cluster in each graph represents a male subject with a baseline BMI of 27.8 kg/m^2^). During high-calorie feeding, L4 vertebral BMAT increased in 69.2% of men, VAT increased in 76.9% of men, and SAT increased in 100% of men (**A**). During fasting, L4 vertebral BMAT increased in 92.3% of men, VAT decreased in 76.9% of men, and SAT decreased in 84.6% of men (**B**). During the 2-week stabilization period, L4 vertebral BMAT decreased in 100% of men, VAT decreased in 84.6% of men, and SAT decreased in 38.5% of men (**C**). Compared with baseline, at the end of the study, L4 vertebral BMAT decreased in 46.2% of men, VAT decreased in 53.8% of men, and SAT decreased in 61.5% of men (**D**).

BMAT at the L4 vertebra also increased significantly during the high-calorie visit (median increase [IQR]: 6.7% [–1.7%, 40.2%], *P* = 0.02) (Table 2, Figure 2A, and Figure 3A). There was an increase in BMAT at the femoral diaphysis (mean change: 8.2% ± 3.2%, *P* = 0.08), but this increase was not statistically significant (Table 2). Correlations between changes in body composition parameters and changes in BMAT during the high-calorie visit are listed in Table 3. Similar to the association observed at baseline, percentage change in lean mass was positively associated with percentage change in BMAT at the femoral diaphysis (*R* = 0.44, *P* = 0.04); there were no other significant associations between changes in BMAT and changes in body composition parameters during the high-calorie visit. Although the baseline BMI range of our study subjects was narrow (BMI range: 23.3–27.9 kg/m^2^, there was a trend toward an inverse association between percentage change in BMAT at the femoral metaphysis and baseline BMI during the high-calorie visit (rho = –0.43, *P* = 0.05). There were no other associations observed between baseline BMI and changes in BMAT during the high-calorie visit.

Comparing changes in the men and women during the high-calorie visit, there were no significant differences in changes in weight, SAT, VAT, or BMAT at any site between men and women (Table 4), whereas there were significant differences in body composition parameters measured by DXA between the groups (Table 4). Changes in VAT and SAT during the high-calorie visit were not correlated with changes in BMAT during the high-calorie visit in either men or women, but in women, the percentage change in SAT during the high-calorie visit was inversely associated with percentage change in BMAT at the femoral metaphysis during the 2-week stabilization period (rho = –0.73, *P* = 0.02), suggesting that greater increases in SAT during the high-calorie visit were associated with lesser increases in BMAT at the femoral metaphysis during the 2-week stabilization period. Similarly, in men, percentage change in SAT during the high-calorie visit was inversely associated with percentage change in L4 BMAT during the 2-week stabilization period (*R* = –0.80, *P* = 0.0009), again suggesting that the greater the increase in SAT with high-calorie feeding, the greater the decrease in L4 BMAT in the 2 weeks after the high-calorie visit (2-week stabilization period). In contrast, in men, percentage change in VAT during the high-calorie visit was positively associated with percentage change in BMAT at the femoral metaphysis during the 2-week stabilization period (*R* = 0.60, *P* = 0.03), potentially suggesting a very different relationship between BMAT and VAT as compared with BMAT and SAT in response to high-calorie intake. In men, there was also a significant positive association between percentage change in lean mass and percentage change in BMAT at the femoral diaphysis in response to high-calorie feeding (*R* = 0.59, *P* = 0.03).

#### Fasting visit.

During the fasting visit, body weight decreased a mean of –8.8% ± 0.3% (*P* < 0.0001; Table 2 and Figure 1B). Similarly, SAT (median decrease: –11.2% [–16.8%, –9.5%], *P* < 0.0001), trunk fat (mean decrease: –9.7% ± 0.9%, *P* < 0.0001), extremity fat (mean decrease: –4.9% ± 0.8%, *P* < 0.0001), and lean mass (mean decrease: –9.8 ± 0.4%, *P* < 0.0001) decreased significantly (Table 2). VAT decreased but not significantly (mean decrease: –5.4% ± 4.0%, *P* = 0.06; Table 2).

BMAT at the L4 vertebra also increased in response to fasting (mean increase: 8.1 ± 2.6%, *P* = 0.01; Table 2, Figure 2B, and Figure 3B). There was a significant inverse association between percentage change in weight and percentage change in femoral metaphysis BMAT during the fasting visit (*R* = –0.53, *P* = 0.009; Table 5) but no other significant associations between percentage change in BMAT and percentage change in body composition parameters in the group as a whole. There was no association between baseline BMI and change in BMAT at any site during the fasting visit.

Comparing changes between men and women during the fasting visit, men gained significantly more BMAT at the L4 vertebra (men: 13.5% ± 3.4 versus women: 1.1% ± 3.1, *P* = 0.01), whereas women gained significantly more BMAT at the femoral metaphysis (men: –6.0% ± 5.9 versus women: 20.5% ± 9.2, *P* = 0.03). Women, who had gained more lean mass than men during the high-calorie visit, similarly lost significantly more lean mass during the fasting visit as compared with men (Table 4). There were no significant differences in percentage change in SAT or VAT between women and men.

In women, there was a strong inverse association between percentage change in weight and percentage change in BMAT at the femoral metaphysis (*R* = –0.95, *P* < 0.0001) during the fasting visit. Therefore, the greater the drop in weight with fasting, the greater the increase in BMAT at the femoral metaphysis in women. In contrast to what was observed in men in response to high-calorie feeding (a positive association between change in BMAT and change in lean mass), percentage change in L4 BMAT was inversely associated with change in lean mass in women (*R* = –0.73, *P* = 0.02) with fasting. This difference could potentially be due to (a) a difference in the association between BMAT and lean mass between the sexes, (b) a difference in the association between BMAT and lean mass in the setting of nutrient sufficiency versus nutrient deficiency, or (c) a difference in the association between BMAT at a more proximal location (L4 vertebra) versus a more peripheral BMAT depot (femoral diaphysis). Changes in VAT and SAT during the fasting visit were not correlated with changes in BMAT during the fasting visit in either men or women.

#### Two-week stabilization period.

Body weight decreased a mean of –2.7% ± 0.4% (*P* < 0.0001) during the 2-week stabilization period, during which participants were instructed to resume their normal diet at home after the high-calorie visit (Table 2 and Figure 1C); there were no a priori goals with respect to change in weight for this period. Changes in body weight from baseline (start of the high-calorie visit) to the end of the 2-week stabilization period are shown in Figure 1D; all but 1 study participant was at a higher weight as compared with baseline at the end of the stabilization period. SAT did not change significantly during the 2-week stabilization period (*P* = 0.19), but VAT decreased a median of –11.4% [–28.4%, 11.7%] (*P* = 0.04; Table 2). Lean mass also decreased significantly during the 2-week stabilization period (mean decrease: –3.8% ± 0.6%, *P* < 0.0001; Table 2).

There were significant decreases in BMAT at the L4 vertebra and femoral diaphysis during the 2-week stabilization period (Table 2). BMAT at the L4 vertebra decreased a mean of –19.3% ± 2.6% (*P* < 0.0001; Figure 2C and Figure 3C), and BMAT at the femoral diaphysis decreased a mean of –10.7% ± 3.5% (*P* = 0.02). Similar to the baseline correlation, percentage change in lean mass was positively associated with percentage change in L4 BMAT (*R* = 0.41, *P* = 0.0496; Table 6). Although both VAT and BMAT decreased significantly during this period, there was not a significant association between change in VAT and change in BMAT, but there was a significant positive association between percentage change in trunk fat and percentage change in L4 BMAT during this stabilization period (*R* = 0.42, *P* = 0.045). There were no other significant associations between changes in body composition and changes in BMAT during the 2-week stabilization period, but percentage change in L4 BMAT during this period was positively associated with baseline BMI (rho = 0.59, *P* = 0.003). Baseline BMI was not associated with changes in femoral BMAT during this stabilization period.

**Figure 4:**
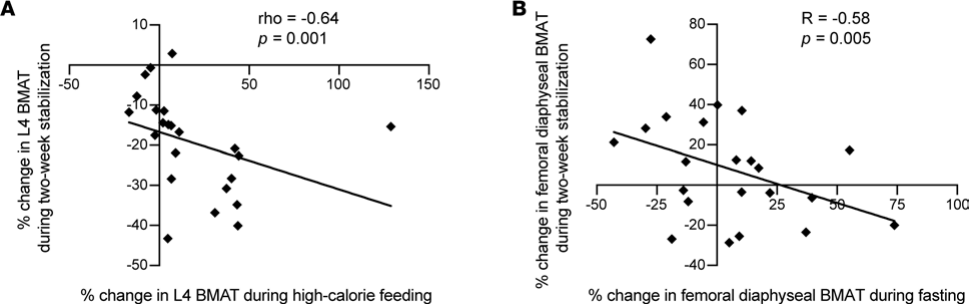
Changes in BMAT during the 2-week stabilization period were associated with changes during both the high-calorie visit and the fasting visit. Percentage change in L4 BMAT during the 2-week stabilization period was significantly associated with percentage change in L4 BMAT during the high-calorie visit (rho = –0.64, *P* = 0.001) (**A**). Percentage change in femoral diaphyseal BMAT during the 2-week stabilization period was significantly associated with percentage change in femoral diaphyseal BMAT during the fasting visit (*R* = –0.58, *P* = 0.005) (**B**).

Changes in BMAT during the 2-week stabilization period were significantly associated with changes in BMAT during the high-calorie visit and/or changes in BMAT during the fasting visit. Percentage change in BMAT at the L4 vertebra during the 2-week stabilization period was inversely associated with percentage change in BMAT at the L4 vertebra during the high-calorie visit (rho = –0.64, *P* = 0.001; Figure 4A). Percentage change in BMAT during the fasting visit at the femoral diaphysis was also inversely associated with (*R* = –0.58, *P* = 0.005) percentage change in BMAT at the femoral diaphysis during the 2-week stabilization period (Figure 4B). There was also a trend toward an inverse association between percentage change in BMAT at the femoral metaphysis during the fasting visit and during the 2-week stabilization period (*R* = –0.41, *P* = 0.06).

When comparing changes in women versus men, there were no significant differences with respect to change in BMAT, SAT, VAT, weight, or body composition parameters as measured by DXA during the 2-week stabilization period (Table 4). Changes in VAT and SAT during the 2-week stabilization period were not correlated with changes in BMAT during this period in men or women. In women, percentage change in trunk fat during the 2-week stabilization period was positively associated with percentage change in BMAT at the femoral metaphysis during this period (*R* = 0.72, *P* = 0.03). Baseline BMI was also significantly and positively associated with percentage change in L4 BMAT in both women (*R* = 0.65, *P* = 0.04) and men (rho = 0.56, *P* = 0.046).

### Changes in peripheral blood hormonal parameters with high-calorie feeding, with fasting, and during the 2-week stabilization period

#### High-calorie visit.

Leptin (*P* < 0.0001), adiponectin (*P* < 0.0001), and IGF-1 (*P* < 0.0001) all increased significantly during the high-calorie visit, whereas ghrelin, an orexigenic hormone, decreased by a mean of –28.9% ± 4.7% (*P* < 0.0001) and IGF-BP2 decreased by a median of –41.3% [–63.4%, –18.1%] (*P* < 0.0001) (Table 2). With respect to bone turnover markers, CTX, a marker of bone resorption, decreased significantly during the high-calorie visit (median change: –20.6% [–51.5%, –8.8%], *P* = 0.002), whereas there was no significant change in P1NP (*P* = 0.75), a marker of bone formation (Table 2). Inflammatory markers TNF-α and CRP both increased significantly in response to high-calorie feeding. TNF-α increased a mean of 20.3% ± 4.7% (*P* = 0.0005), and CRP increased a median of 135.4% [27.3%, 239.6%] (*P* = 0.002).

**Figure 5:**
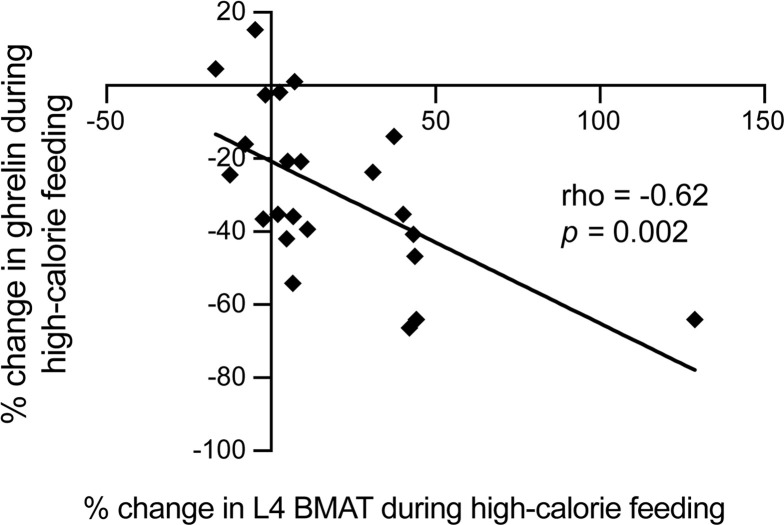
Percentage change in L4 BMAT was inversely associated with percentage change in ghrelin during the high-calorie visit (rho = –0.62, *P =* 0.002). When the subject who gained more L4 BMAT than the other subjects (128.8%) was excluded from the analysis, the results remained significant (rho = –0.57, *P* = 0.006).

As part of an exploratory analysis to determine hormonal predictors of BMAT, we investigated univariate associations between changes in BMAT with high-calorie feeding and changes in adipokines, bone turnover markers, inflammatory markers, and changes in G-CSF levels, given recent data demonstrating suppression of marrow adipocytes by G-CSF in a murine model (ref. 7 and Supplemental Table 7). We found a significant inverse association between percentage change in ghrelin and percentage change in BMAT at the L4 vertebra (rho = –0.62, *P* = 0.002; Figure 5), in contrast to the positive baseline association between BMAT and ghrelin observed in male study participants. There was also a significant inverse association between percentage change in BMAT at the L4 vertebra and percentage change in IGF-BP2 (rho = –0.44, *P* = 0.04; Supplemental Table 7), again in contrast to the observed baseline association between BMAT and IGF-BP2 in female study participants.

#### Fasting visit.

Leptin (*P* < 0.0001) and adiponectin (*P* < 0.0001) decreased significantly in response to fasting. This drop in adiponectin is consistent with prior human data demonstrating a decrease in adiponectin levels with fasting (14). In contrast, in a mouse model of caloric restriction, adiponectin levels increased (15), underscoring important differences between murine and human models. Ghrelin, which decreased in response to high-calorie feeding, also decreased in response to fasting (mean decrease –64.1% ± 5.3%, *P* < 0.0001). With respect to bone turnover markers, CTX increased (median increase: 77.8% [43.7%, 133.8%], *P* < 0.0001) and P1NP decreased (mean decrease: –43.4% ± 2.1%, *P* < 0.0001) significantly in response to fasting. CRP, which increased in response to high-calorie feeding, also increased in response to fasting (median increase: 100% [38.3%, 277.0%], *P* = 0.0003), and GDF15, another inflammatory marker, also increased in response to fasting (*P* = 0.0005; Table 2). These findings are consistent with the increased inflammatory response observed in prolonged human fasting (16). Changes in adipokines, bone turnover markers, or inflammatory markers did not predict changes in BMAT during the fasting visit (Supplemental Table 8).

#### Two-week stabilization period.

Changes in adipokines, bone turnover markers, and inflammatory markers during the 2-week stabilization period are listed in Table 2. Ghrelin and FGF21 levels significantly increased during the 2-week stabilization period (median increase in ghrelin: 39.6% [6.9%, 96.9%], *P* = 0.0001; median increase in FGF21: 64.1% [–9.7%, 306.7%], *P* = 0.02). In contrast to the observed baseline association in female study participants, there was a positive association between percentage change in FGF21 and percentage change in BMAT at the femoral diaphysis (rho = 0.48, *P* = 0.03). Consistent with data in a murine model demonstrating suppression of marrow adipocytes in response to endogenous G-CSF (7), percentage change in G-CSF was inversely associated with percentage change in BMAT at the femoral diaphysis (rho = –0.52, *P* = 0.01; Supplemental Table 9).

### Changes in marrow molecular and biochemical parameters with high-calorie feeding and fasting

We performed whole bone marrow aspirations on 9 subjects at baseline and on the final day of the high-calorie visit and 11 distinct participants on the baseline day and final day of the fasting visit. This led to paired samples (*n* = 9 for high-calorie and *n* = 11 for fasting) from the same individual undergoing 1 of the 2 nutritional challenges but not both. For marrow serum markers, we assessed adiponectin, IL-6, and resistin levels paired before and after the nutritional intervention. Peripheral blood adiponectin levels increased after 10 days of the high-calorie diet and decreased significantly after the fasting intervention (Table 2), as previously reported in human fasting (14), but these changes were not reflected in the marrow sera (data not shown). Resistin, a ligand for TLR4 and the inflammasome, is known to increase in adipose tissue with high-calorie feeding. In humans, most resistin is made by macrophages, and higher levels have been reported in marrow compared with human sera (17). During the high-calorie visit, resistin levels by ELISA rose significantly in the marrow sera from a mean (± SEM) of 26.7 ± 3.1 ng/mL to 37.7 ± 5.3 ng/mL (*P* < 0.05), but this rise was not reflected in peripheral blood levels (data not shown). In addition, there was a nonsignificant decline in marrow sera resistin 10 days after fasting but again no change in peripheral blood resistin with fasting. IL-6 did not change in the marrow sera of individuals undergoing the high-calorie or fasting intervention (data not shown).

**Figure 6:**
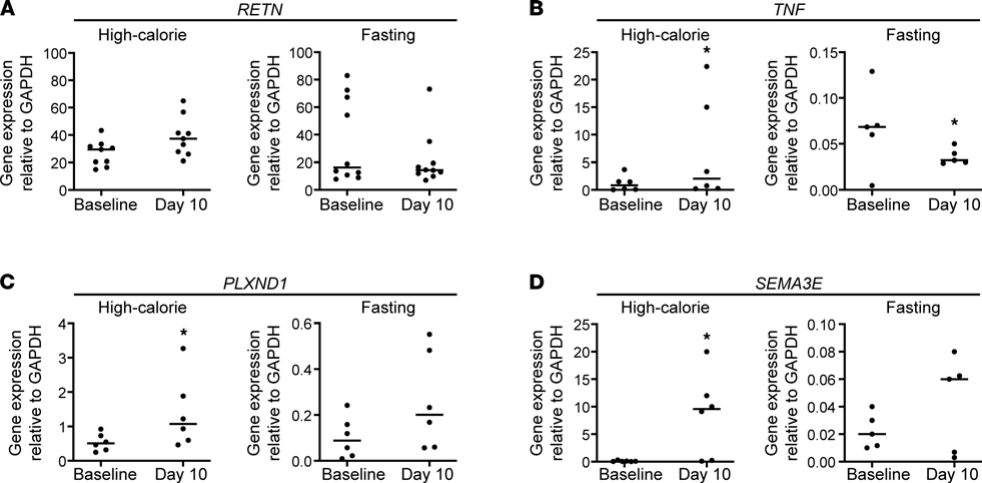
Gene expression was measured by real-time quantitative PCR of mature adipocytes in paired samples at baseline and day 10 of high-calorie feeding and baseline and day 10 of fasting. (**A**) *RETN* (resistin); (**B**) *TNF* (TNF-α); (**C**) *PLXND1* (Plexin D1); (**D**) SemaphoreE3 (*SEMA3E*). Paired-sample 2-tailed *t* test (*P < 0.05). The mRNA expression of each gene was normalized to GAPDH.

We next asked whether mature marrow adipocytes exhibited expression pattern changes in response to high-calorie or fasting interventions, again using paired samples obtained with marrow aspiration (Figure 6). First, unlike the marrow sera, expression of resistin mRNA was not enhanced with high-calorie feeding (Figure 6A); this almost certainly is because resistin is made primarily by macrophages in humans and adipocytes in rodents (17). Among fasting individuals, there was also no change in resistin in adipocyte gene expression from paired samples of adipocytes. Plexin D1, SEMA3E, and TNF-α mRNA all were significantly increased in marrow adipocytes after the 10-day high-calorie intervention (Figure 6, B–D). In contrast, TNF-α mRNA in adipocytes declined significantly with fasting (Figure 6B), and the other inflammatory gene expression markers did not show significant changes.

## Discussion

We show that BMAT increased significantly during both an acute high-calorie intervention and a fasting intervention, resulting in a significant increase and decrease in weight, respectively. We also demonstrate that BMAT decreased significantly during a 2-week stabilization period following the acute high-calorie intervention, during which there was a statistically significant but clinically minimal decrease in body weight. Importantly, we now demonstrate that high-calorie diet rapidly induced a marrow inflammatory immune response, similar to what is observed in peripheral adipose depots, but not found with fasting. These data underscore the concept that BMAT is a dynamic adipose tissue depot that responds to nutrient flux in a rapid and reversible fashion, reflecting its unique location and function. However, the functional differences in the marrow between BMAT during fasting and BMAT during high-calorie feeding await further translational studies.

Marrow adipocytes reside in the bone marrow microenvironment along with hematopoietic and osteoblast progenitors, and therefore, a number of studies investigating the association between BMAT and bone mineral density (BMD) have been performed. These studies demonstrate that BMAT, which has been shown to represent approximately 13% of total adipose tissue in healthy individuals (15), is inversely associated with BMD in multiple populations, including individuals with anorexia nervosa (2, 3). In women with anorexia nervosa, this association between increased BMAT and low BMD is particularly striking because other adipose tissue depots, including the visceral and subcutaneous depots, are decreased compared with normal-weight individuals (1, 2), specifically because the lipid stores are being used as an energy source in a state of chronic starvation. Why BMAT stores would be conserved during starvation is not known, but striking differences between BMAT and other fat depots with respect to lipolytic activity have been shown in both animal studies (18, 19) and human studies (20).

We have previously shown that BMAT appears to behave differently in states of chronic starvation as compared with states of nutritional sufficiency. For example, although BMAT is positively associated with VAT in studies including overweight/obese women (11) and as we confirm with these data, this is in contrast to what is observed in studies including only normal- and low-weight women (5). This suggests that BMAT may serve as an ectopic fat depot, similar to the role of VAT, in overweight/obese women but may serve a different function in states of starvation, when lipid stores may be actively used as an energy source. The fact that BMAT increased during both the high-calorie and the fasting interventions supports this concept that BMAT may serve different functions in states of caloric excess versus caloric deprivation.

Intriguingly, the largest change in BMAT was not during high-calorie intake or during fasting but instead during the 2-week period when subjects resumed their normal diet after high-calorie feeding. During this 2-week period, there was a 17.8% drop in L4 vertebral BMAT in women and a 20.4% drop in L4 vertebral BMAT in men. Whether this drop in BMAT reflects a redistribution of lipid stores after a period of acute weight gain warrants further study. Our data also support the concept of region-specific variation in BMAT response to environmental cues. We have previously demonstrated in murine models that distal skeletal BMAT sites are less responsive to systemic challenges compared with proximal skeletal BMAT sites (21). Here we show that as observed in mice, human bone marrow adipocytes respond differently to nutrient challenges depending on their location. We also demonstrate that there may be region-specific variation in BMAT response to environmental cues based on sex. We observed significant differences in women versus men during fasting with respect to site of BMAT accumulation, with men accumulating significantly more BMAT in the L4 vertebra during fasting as compared with women and women accumulating significantly more BMAT at the femoral metaphysis during fasting as compared with men.

Data from marrow aspirates in this study provide a unique perspective on the adipocytic response to nutrient changes. High-calorie feeding induced a proinflammatory response with increases in TNF-α, Plexin D1, and SEMA3E gene expression in mature adipocytes at day 10, as well as a marked increase in marrow serum resistin, a putative measure of macrophage activation. These proinflammatory mediators induce the recruitment and activation of adipose tissue macrophages (ATMs). The activated ATMs secrete proinflammatory cytokines and form the inflammatory circuit, which blocks the insulin action of adipocytes and leads to insulin resistance. On the other hand, no markers of inflammation were noted in the fasting aspirates after 10 days (Figure 6). Taken together, we postulate that much like peripheral adipose depots during high-fat feeding, marrow adipocytes are accompanied by an inflammatory response, including a rise in marrow serum resistin, likely from macrophages, that could contribute to long-term sequelae from high-calorie diets, including the metabolic syndrome. Moreover, these studies suggest that not every type of marrow adiposity is functionally the same nor is its relationship to peripheral adipose depots, as noted from the fasting data.

The timing of caloric excess or deprivation (chronic vs. acute) plays a major role in hormonal responses. We demonstrate that although ghrelin levels decreased in response to high-calorie feeding, as would be expected, given ghrelin’s role as an orexigenic hormone, ghrelin levels also decreased significantly with fasting. This unexpected finding contrasts with what is observed in a state of chronic caloric deprivation, anorexia nervosa, in which ghrelin levels are elevated (22–24). One important limitation is that we only measured total ghrelin levels and not acylated and desacyl ghrelin; therefore, whether these changes in total ghrelin levels are due to differences in acylated levels versus desacyl levels will be critical to determine in future studies. In a rodent model, ghrelin has been shown to promote marrow adipogenesis (13) but, importantly, likely through a receptor other than the growth hormone secretagogue receptor 1a to which acylated ghrelin is known to bind. Based on these data, we hypothesized that changes in ghrelin would be positively associated with changes in BMAT in this study but observed the opposite. Change in ghrelin was inversely associated with BMAT during the high-calorie intervention. Whether this is due to a divergent association between ghrelin and BMAT in humans as compared with rodents or differential levels of acyl versus desacyl ghrelin levels in high-calorie feeding versus fasting remains to be determined.

In murine models, FGF21, like ghrelin, is a hormonal determinant of BMAT. FGF21 is a hormone induced during starvation in murine models as well as in humans and is a mediator of ketogenesis in murine models but not in humans (14, 25, 26). FGF21 has additional metabolic properties, including as a mediator of insulin-independent glucose uptake (27). FGF21-transgenic mice have higher bone marrow adipocyte number and area (12), and although our baseline, cross-sectional data in female study participants demonstrated an inverse association between FGF21 and BMAT, our longitudinal data demonstrated a positive association between changes in FGF21 and BMAT during the 2-week stabilization period, consistent with these animal data. With the high-calorie intervention, FGF21 levels significantly decreased and then subsequently significantly increased during the 2-week stabilization period. Although mean levels of FGF21 increased with fasting, this change was not significant, in contrast to our prior data demonstrating significant increases in FGF21 after a 10-day fast (14). This difference may be due to metabolic changes induced by the high-calorie intervention preceding the fast in this study.

In conclusion, we demonstrate in an acute 10-day high-calorie feeding protocol followed by a 10-day fasting protocol that BMAT increased in response to both interventions, but the pathophysiology and cues for these changes likely differ. These data support the concept that nutrient flux rather than body weight is an important determinant of BMAT. A limitation of our study is that the fasting intervention followed the high-calorie protocol for all study subjects. Therefore, changes that we observed during fasting were likely in part modified by changes during an acute high-calorie intervention. Nevertheless, these data demonstrating changes in body composition, appetite-regulating hormones, bone turnover markers, and inflammatory markers after a high-calorie intervention and a fasting intervention will provide a resource for future human studies. This work also demonstrated a number of observations in humans, including the remarkable changes in BMAT with acute changes in nutrient intake, as well as the significant decrease in ghrelin after both a high-calorie intervention as well as a fasting intervention. Finally, bone marrow aspirates during acute nutrient fluxes, which have not been reported previously to our knowledge, revealed a remarkable difference in the marrow environment in response to a high-calorie diet compared with fasting.

## Methods

### Subjects

We studied 23 individuals (10 women and 13 men). All individuals (mean age and age range: 33.3 years and 22–44 years) were normal weight or overweight (BMI range: 23.3–27.9). No subject had a history of diabetes mellitus or a history of an eating disorder, and none of the participants were taking chronic medications. All women were premenopausal and had a history of regular menstrual cycles, and none had used exogenous estrogen within 3 months of her baseline visit. An additional female participant enrolled in the study (age at enrollment: 31 years and BMI at enrollment: 24.8) but was not able to comply with the high-calorie protocol and did not gain weight during the first study visit (%change in weight during high-calorie protocol: –0.3%); therefore, her data have not been included in the analyses.

### Study protocol

All subjects were admitted to the TCRC at the Massachusetts General Hospital for 2 inpatient study visits totaling up to 20 days. Participants were initially admitted for a 10-day high-calorie visit, during which time they met with a bionutritionist, who calculated their caloric needs for 7% weight gain based on body weight using the Mifflin St Jeor equation with an activity factor of 1.3 (28, 29). Participants were permitted to select menu items with a macronutrient content consisting of 45% to 55% carbohydrates, 30% to 40% fat, and no more than 25% protein. After completion of the high-calorie protocol, subjects were discharged home to resume their normal diet for 13 to 18 days (median: 15 days with range: 14–18 days). The interval for this 2-week stabilization period ranged from 13 to 18 days in order to accommodate study participant schedule requests as well as to appropriately time the imaging scans for the second inpatient visit, as some scans could not be performed on weekends. Subjects were subsequently readmitted for a second inpatient visit (fasting visit), during which subjects did not consume any calories for 7 to 10 days but were permitted to drink water ad libitum and received a multivitamin containing 400 IU of cholecalciferol daily as well as 20 mEq of potassium chloride. Subjects were weighed on an electronic scale each inpatient morning and were blinded to their weights. On the first day (baseline day) of each study visit and the final high-calorie inpatient day and final fasting day, subjects underwent radiologic imaging (outlined below), and blood was drawn for laboratory studies (fasting). Height was measured on the first inpatient day of the high-calorie visit and was the average of 3 readings on a single stadiometer. Overall changes in body composition parameters between the start of the study and the end of the study are shown in Figure 2D and Figure 3D. After subjects completed the 10-day fast, subjects were instructed on refeeding by a bionutritionist and monitored overnight as caloric intake was resumed. Nine subjects (*n* = 6 men and *n* = 3 women) underwent bone marrow aspiration (described below) at baseline and on the final day of the high-calorie visit, and 11 subjects (*n* = 6 men, *n* = 5 women) who did not undergo bone marrow aspiration during the high-calorie visit had a bone marrow aspiration on both the baseline day and final day of the fasting visit.

### Radiologic imaging

#### Dual-energy x-ray absorptiometry.

All subjects underwent DXA on the baseline day of each inpatient visit and on the final day of the high-calorie visit and the final fasting day. Body composition parameters including trunk fat, extremity fat, and lean mass were measured by DXA. Coefficients of variation (CV) for DXA have been reported to be no more than 1.1% for total body lean mass and range from 2.4% to 6.7% for trunk fat and extremity fat measurements (30).

#### Magnetic resonance imaging and ^1^H-MRS.

A single axial magnetic resonance imaging (Siemens Trio, 3T, Siemens Medical Systems) slice (at the level of the L4 vertebra) was used to measure abdominal SAT and VAT.

^1^H-MRS was performed to determine lipid content in the marrow (Siemens Trio, 3T, Siemens Medical Systems). For the L4 vertebra, a voxel measuring 15 × 15 × 15 mm (3.4 mL) was placed within the L4 vertebral body. Single-voxel ^1^H-MRS data were acquired using point-resolved spatially localized spectroscopy (PRESS) pulse sequence without water suppression with the following parameters: echo time of 30 ms, repetition time of 3000 ms, 8 acquisitions, 1024 data points, and receiver bandwidth of 2000 Hz. For the femur, a voxel measuring 12 × 12 × 12 mm (1.7 mL) was positioned within the metaphysis at the inter-trochanteric region, and single-voxel ^1^H-MRS using the same non–water-suppressed PRESS pulse sequence was performed. This process was repeated with voxel placement in the mid-diaphysis. Automated procedures for optimization of gradient shimming and transmit and receive gain were used. We have reported the CV for marrow fat quantification in a population of premenopausal women in our institution to be 3% (4).

Fitting of the ^1^H-MRS data was performed using LCModel software (version 6.1-4A) (Stephen Provencher). Data were transferred from the scanner to a Linux workstation, and metabolite quantification was performed using eddy current correction and water scaling. A customized fitting algorithm for bone marrow analysis provided estimates for all lipid signals combined (0.9, 1.3, and 2.3 ppm). LCModel bone marrow lipid estimates were automatically scaled to unsuppressed water peak (4.7 ppm) and expressed as lipid/water ratio. ^1^H-MRS assessment of the L4 vertebra for a subject before and after both the high-calorie feeding and fasting visits is illustrated in Supplemental Figure 1.

### Bone marrow aspiration

Subjects were placed in the prone position, and the posterior iliac crest was sterilely prepared using the ChloraPrep applicator (CareFusion). Two percent lidocaine was used to infiltrate the subcutaneous tissues and periosteum. Using an 11G OnControl bone marrow biopsy device (Teleflex Medical), a bone marrow aspirate was obtained using standard protocols (31) and collected in an EDTA tube (adipocyte collection) and tiger-top tube (marrow sera collection). Then, 5 mL of PBS was added to the aspirate collected in the EDTA tube, which was then spun at 377*g* for 8 minutes at 4°C. The top layer was then transferred to a microfuge tube; TRIzol reagent was added (1:1; Thermo Fisher Scientific) and the samples were stored at –80°C. Samples in the tiger-top tubes were spun at 2054*g* for 10 minutes at 4°C. The top layer was aliquoted and stored at –80°C. The majority of cells were mature adipocytes, but the possibility that a small number of adherent stromal vascular cells were isolated with floated adipocytes cannot be excluded.

### Biochemical assessment

#### Peripheral blood.

Serum leptin, adiponectin, FGF21, G-CSF, IL-6, TNF-α, resistin, and GDF15 were measured by human DuoSet ELISA (R&D Systems, Bio-Techne). For leptin, the intra-assay CV was 3.2% and the inter-assay CV: 4.4%. The intra-assay CV for adiponectin was 3.9% and the inter-assay CV: 6.5%. For FGF21, the intra-assay CV was 3.4% and the inter-assay CV: 7.5%. The intra-assay CV for G-CSF was 1.9% with an inter-assay CV of 3.7%. For IL-6, the intra-assay CV was 2.6% and the inter-assay CV: 4.5%. For TNF-α, the intra-assay CV was 2.0% and the inter-assay CV was 6.5%. The intra-assay CV for resistin was 5.0%, The intra- and inter-assay CV for resistin were less than 9.2%. The intra-assay CV for GDF15 was 2.3% with an inter-assay CV of 5.4%. Ghrelin was measured by ELISA (Thermo Fisher Scientific) with an intra-assay CV of 6.0% and an inter-assay CV of 8.5%. P1NP, a marker of bone formation, and CTX, a marker of bone resorption, were measured by a luminescent immunoassay analyzer (ISYS Analyzer, Immunodiagnostics Corporation). For P1NP, the intra-assay CV was 3.0% and the inter-assay CV was 5.0%. For CTX, the intra-assay CV was 3.2% and the inter-assay CV was 6.2%. IGF-BP2 was measured by ELISA (ALPCO) with a detection limit of 0.2 ng/mL, and intra- and inter-assay CV were both less than 10%. IGF-1 was measured in a clinical laboratory (Quest Diagnostics Nichols Institute). Insulin and CRP were also measured by a clinical laboratory (Quest Diagnostics), and glucose was measured as previously described (32). HOMA-IR was calculated using the following equation: (glucose in mg/dL × insulin in uIU/mL)/405.

#### Bone marrow sera.

After the bone marrow aspiration, PBS 5 cc was added to the aspirate (also containing cells) and collected in the EDTA tube. Bone marrow aspirate was then spun at 377*g* for 8 minutes at 4°C. The remaining components were divided into 3 compartments: compartment 1, pellet, which contained the stromal vascular fraction; compartment 2, the fluid between the pellet and the top layer, which we called marrow serum because of its appearance; and compartment 3, the top layer of floated cells. Measurements of adiponectin, IL-6, and resistin were performed on marrow sera using the conventional sandwich ELISA kits (DuoSet, R&D Systems, Bio-Techne) according to the manufacturer’s instructions. The detection limits were 1 ng/mL, 5.0 pg/mL, and 10 pg/mL, respectively.

### Real-time quantitative PCR analysis of gene expression

Total RNA was purified from mature adipocytes using the RNeasy Micro Kit, which included a DNase I treatment step as per manufacturer’s instructions (QIAGEN). First-strand cDNA was synthesized from 0.5 μg total RNA using the iScript cDNA Synthesis Kit (Bio-Rad). Primer sequences used for real-time quantitative PCR are listed in Supplemental Table 10. Quantitation of human transcript gene expression was performed on the CFX96 (Bio-Rad). The relative fold differences in transcript expression were calculated by adapting the 2–ΔCt × 100 formula (33).

### Statistics

Statistical analysis was performed using JMP Pro 13.0 (SAS Institute) software. Normally distributed data were reported as means ± SEM and compared using the 2-tailed *t* test. Non-normally distributed data were reported as median [interquartile range] and were compared using the Wilcoxon test. Paired-sample 2-tailed *t* test or Wilcoxon signed-rank test (if data were non-normally distributed) was used to compare changes in body composition parameters and hormone levels between the baseline day and final day of each study visit and the final day of the high-calorie visit and the first day of the fasting visit (to assess changes during the 2-week stabilization period). For normally distributed data, Pearson’s correlation coefficients (represented by *R*) were calculated to assess univariate relationships. For non-normally distributed data, Spearman’s coefficients (represented by rho) were calculated to assess univariate relationships. A *P* value of less than 0.05 was considered significant.

### Study approval

The study was approved by the Partners HealthCare Institutional Review Board (Boston, Massachusetts, USA) and complied with the Health Insurance Portability and Accountability Act guidelines. Written informed consent was obtained from all subjects.

## Author contributions

PKF, MAB, HL, OAM, MCH, CJR, and AK participated in the study design. PKF, MAB, WZ, XZ, ATF, MR, SPP, TMH, HL, and EKO conducted the human experiments and/or were responsible for acquisition of data and their analysis. GPP and CJR were responsible for the design and conduct of the bone marrow aspirate analyses. PKF and CJR contributed to the writing of the manuscript. All authors critically reviewed and approved the manuscript.

## Supplementary Material

Supplemental data

Trial reporting checklists

ICMJE disclosure forms

## Figures and Tables

**Table 1:**
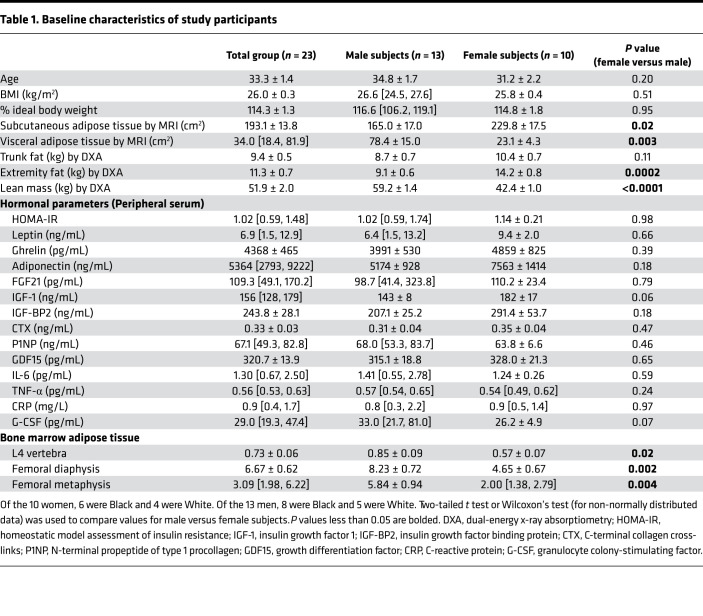
Baseline characteristics of study participants

**Table 2:**
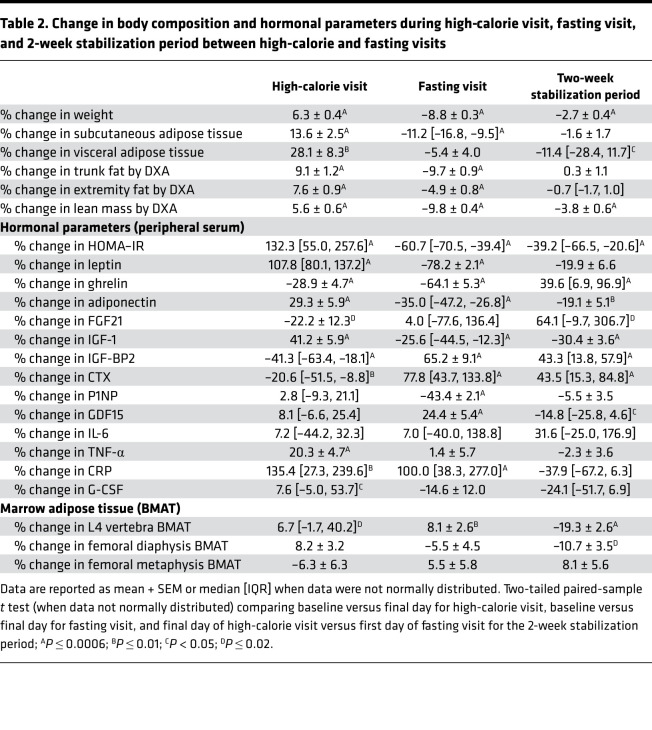
Change in body composition and hormonal parameters during high-calorie visit, fasting visit, and 2-week stabilization period between high-calorie and fasting visits

**Table 3:**
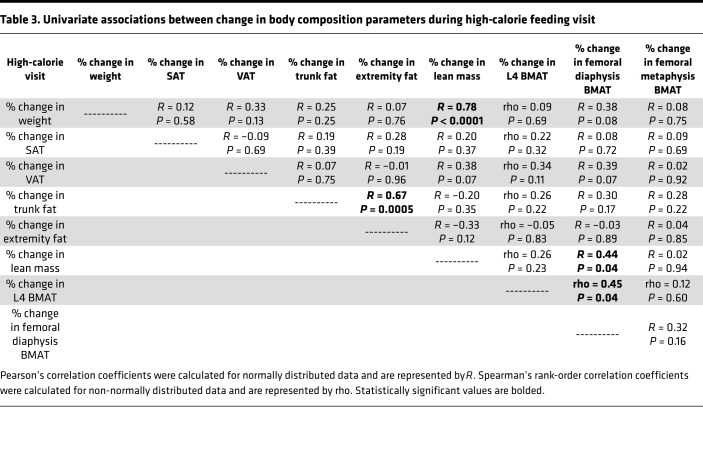
Univariate associations between change in body composition parameters during high-calorie feeding visit

**Table 4:**
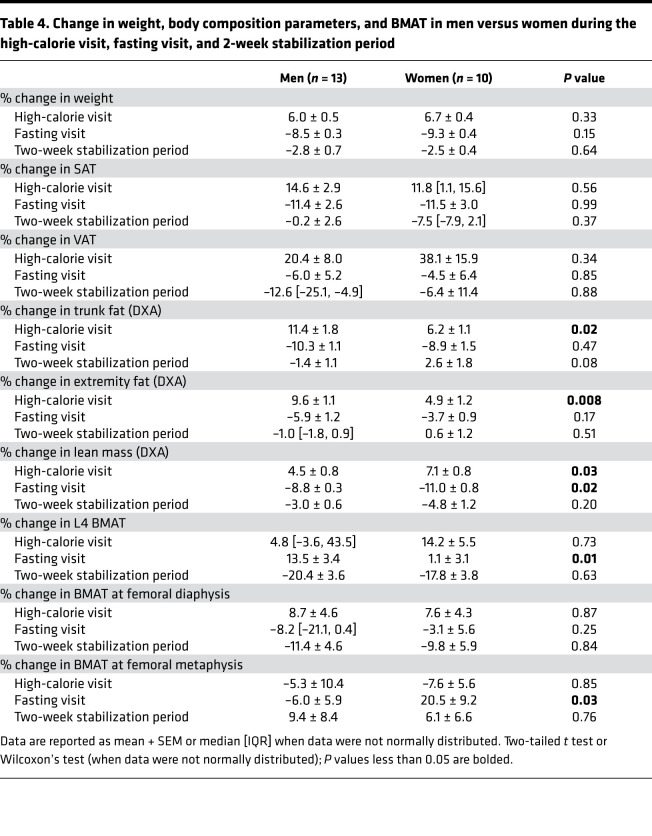
Change in weight, body composition parameters, and BMAT in men versus women during the high-calorie visit, fasting visit, and 2-week stabilization period

**Table 5:**
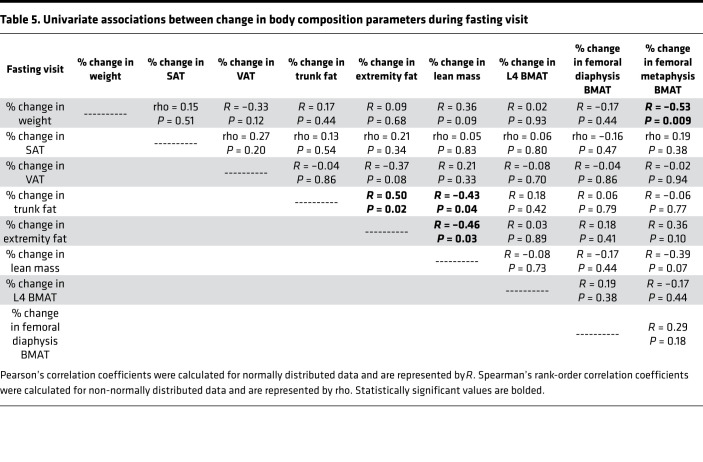
Univariate associations between change in body composition parameters during fasting visit

**Table 6:**
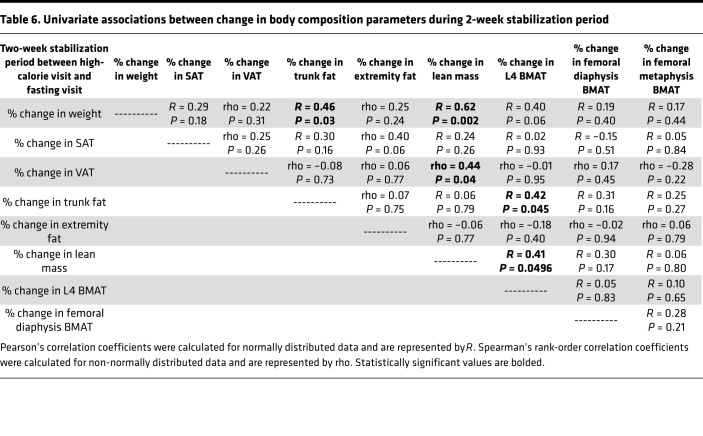
Univariate associations between change in body composition parameters during 2-week stabilization period

## Version 1 05/11/2021

In-Press Preview

## Version 2 06/22/2021

Electronic publication
