# Supplementary material for: The dynamics of human bone marrow adipose tissue in response to feeding and fasting
Source: JCI Insight. 2021 Jun 22;6(12):e138636. doi: 10.1172/jci.insight.138636 (PMC8262500; doi:10.1172/jci.insight.138636)
Supplement: Supplemental data [file jciinsight-6-138636-s105.pdf]

Supplemental Table 1: Baseline correlations between body composition parameters. Pearson correlation coefficients were calculated for normally distributed data and are represented by R. Spearman rank-order correlation coefficients were calculated for non-normally distributed data and are represented by rho.

All subjects	**BMI**	**SAT**	**VAT**	**Trunk fat**	**Extremity fat**	**Lean mass**	**L4 BMAT**	**Femoral diaphysis BMAT**	**Femoral metaphysis BMAT**
**BMI**	----------	**R=0.43 p=0.04**	**rho=0.44 p=0.04**	**R=0.50 p=0.01**	R=0.29 p=0.18	R=0.19 p=0.38	R=0.08 p=0.70	R= -0.26 p=0.23	rho=0.17 p=0.44
**SAT**		----------	rho=0.20 p=0.37	**R=0.83 p<0.0001**	**R=0.70 p=0.0002**	**R= -0.46 p=0.03**	R=0.03 p=0.90	**R= -0.49 p=0.02**	rho= -0.06 p=0.78
**VAT**			----------	rho=0.32 p=0.14	rho= -0.31 p=0.15	**rho=0.50 p=0.02**	**rho=0.57 p=0.004**	rho=0.25 p=0.26	**rho=0.59 p=0.004**
**Trunk fat**				----------	**R=0.64 p=0.0009**	R= -0.26 p=0.23	R=0.19 p=0.39	**R= -0.57 p=0.005**	rho= -0.20 p=0.38
**Extremity fat**					----------	**R= -0.58 p=0.004**	R= -0.24 p=0.27	**R= -0.62 p=0.002**	rho= -0.38 p=0.09
**Lean mass**						----------	**R=0.44 P=0.03**	**R= 0.55 p=0.007**	**rho=0.49 p=0.02**
**L4 BMAT**							----------	R=0.30 p=0.17	**rho=0.55 p=0.008**
**Femoral diaphysis BMAT**								----------	**rho=0.62 p=0.002**
**Femoral metaphysis BMAT**									----------

Supplemental Table 2: Baseline correlations between body composition parameters and hormonal parameters. Pearson correlation coefficients were calculated for normally distributed data and are represented by R. Spearman rank-order correlation coefficients were calculated for non-normally distributed data and are represented by rho.

	HOMA-IR	Leptin	Ghrelin	Adiponectin	FGF21	IGF-1	IGF-BP2	CTX	P1NP	GDF-15	IL-6	TNFα	CRP	G-CSF
**BMI**	rho=0.06<br>p=0.78	rho=0.17<br>p=0.44	**R= -0.46<br>p=0.03**	**rho= -0.55<br>p=0.006**	rho= -0.32<br>p=0.15	rho= -0.26<br>p=0.23	**R= -0.47<br>p=0.02**	**R= -0.45<br>p=0.03**	rho= -0.05<br>p=0.83	R= -0.25<br>p=0.25	rho=0.05<br>p=0.82	rho=0.35<br>p=0.10	rho=0.09<br>p=0.68	rho=0.31<br>p=0.15
**SAT (MRI)**	rho=0.28<br>p=0.19	rho=0.34<br>p=0.11	R= -0.10<br>p=0.66	rho= -0.01<br>p=0.96	rho= -0.16<br>p=0.48	rho= -0.09<br>p=0.70	R= -0.15<br>p=0.51	R=0.02<br>p=0.91	rho=0.11<br>p=0.62	R=0.07<br>p=0.74	rho= -0.11<br>p=0.66	rho= -0.19<br>p=0.38	**rho=0.54<br>p=0.01**	rho=0.003<br>p=0.99
**VAT (MRI)**	rho=0.27<br>p=0.21	rho=0.07<br>p=0.74	rho= -0.26<br>p=0.23	rho= -0.34<br>p=0.11	rho= -0.22<br>p=0.34	**rho= -0.53<br>p=0.009**	rho= -0.34<br>p=0.11	rho= -0.02<br>p=0.93	rho=0.19<br>p=0.39	rho= -0.11<br>p=0.60	rho= -0.06<br>p=0.82	rho= -0.14<br>p=0.52	rho=0.41<br>p=0.06	rho=0.22<br>p=0.31
**Trunk fat (DXA)**	rho=0.30<br>p=0.16	rho=0.26<br>p=0.24	R= -0.22<br>p=0.30	rho= -0.03<br>p=0.90	rho= -0.20<br>p=0.37	rho= -0.13<br>p=0.55	R= -0.26<br>p=0.24	R= -0.002<br>p=0.99	rho=0.14<br>p=0.53	R=0.17<br>p=0.43	rho= -0.19<br>p=0.42	rho= -0.03<br>p=0.88	**rho=0.54<br>p=0.01**	rho=0.14<br>p=0.53
**Extremity fat (DXA)**	rho=0.12<br>p=0.58	rho=0.22<br>p=0.31	R=0.01<br>p=0.97	rho=0.07<br>p=0.74	rho= -0.29<br>p=0.19	rho=0.33<br>p=0.12	R= -0.09<br>p=0.70	R= -0.02<br>p=0.93	rho= -0.08<br>p=0.72	R=0.20<br>p=0.36	rho= -0.22<br>p=0.35	rho= -0.10<br>p=0.65	rho=0.22<br>p=0.32	rho= -0.15<br>p=0.49
**Lean mass (DXA)**	rho=0.14<br>p=0.52	rho=0.01<br>p=0.97	R= -0.11<br>p=0.61	rho= -0.37<br>p=0.09	rho=0.23<br>p=0.31	rho= -0.26<br>p=0.24	R= -0.37<br>p=0.09	R= -0.27<br>p=0.21	rho=0.02<br>p=0.94	R= -0.05<br>p=0.83	rho= 0.04<br>p=0.86	**rho=0.43<br>p=0.04**	rho=0.06<br>p=0.78	**rho=0.54<br>p=0.008**
**L4 vertebra BMAT**	rho=0.38<br>p=0.08	rho=0.08<br>p=0.72	R= -0.23<br>p=0.28	rho= -0.24<br>p=0.27	rho= -0.24<br>p=0.29	rho= -0.14<br>p=0.53	R= -0.25<br>p=0.26	R=0.04<br>p=0.87	rho=0.08<br>p=0.73	R=0.20<br>p=0.37	rho=0.03<br>p=0.90	rho=0.09<br>p=0.67	rho=0.07<br>p=0.77	rho=0.08<br>p=0.71
**Femoral diaphysis BMAT**	rho=0.05<br>p=0.80	**rho= -0.43<br>p=0.04**	R=0.25<br>p=0.25	rho= -0.27<br>p=0.21	rho=0.39<br>p=0.07	rho=0.14<br>p=0.53	R=0.07<br>p=0.74	R=0.12<br>p=0.60	rho=0.01<br>p=0.95	R=0.03<br>p=0.88	rho=0.21<br>p=0.36	rho= -0.02<br>p=0.94	rho= -0.17<br>p=0.45	rho=0.13<br>p=0.55
**Femoral metaphysis BMAT**	rho=0.27<br>p=0.23	rho= -0.01<br>p=0.97	rho= -0.13<br>p=0.55	**rho= -0.42<br>p=0.049**	rho= -0.08<br>p=0.72	rho= -0.19<br>p=0.39	rho=0.03<br>p=0.90	rho= -0.16<br>p=0.47	rho=0.02<br>p=0.91	rho=0.09<br>p=0.68	rho=0.31<br>p=0.20	rho=0.01<br>p=0.96	rho=0.17<br>p=0.46	rho=0.14<br>p=0.52

Supplemental Table 3: Baseline correlations between body composition parameters (female subjects). Pearson correlation coefficients were calculated for normally distributed data and are represented by R. Spearman rank-order correlation coefficients were calculated for non-normally distributed data and are represented by rho.

Female subjects	BMI	SAT	VAT	Trunk fat	Extremity fat	Lean mass	L4 BMAT	Femoral diaphysis BMAT	Femoral metaphysis BMAT
**BMI**	----------	R=0.16 p=0.65	R=0.27 p=0.45	R=0.31 p=0.38	R=0.38 p=0.28	R=0.02 p=0.96	R=0.15 p=0.68	R= -0.47 p=0.17	rho=0.03 p=0.93
**SAT**		----------	**R=0.71 p=0.02**	**R=0.68 p=0.03**	R=0.42 p=0.22	R= -0.32 p=0.37	**R=0.66 p=0.04**	R= -0.46 p=0.18	rho= 0.35 p=0.33
**VAT**			----------	R=0.50 p=0.15	R= -0.10 p=0.79	R= -0.30 p=0.39	**R=0.73 p=0.02**	R= -0.26 p=0.47	**rho=0.64 p=0.048**
**Trunk fat**				----------	R=0.54 p=0.11	R=0.32 p=0.37	R=0.63 p=0.05	**R= -0.70 p=0.02**	rho= -0.10 p=0.78
**Extremity fat**					----------	R= -0.02 p=0.95	R=0.23 p=0.52	R= -0.48 p=0.16	rho= 0.08 p=0.83
**Lean mass**						----------	R=0.01 p=0.98	R= -0.06 p=0.87	rho= -0.42 p=0.23
**L4 BMAT**							----------	R= -0.19 p=0.59	rho=0.31 p=0.38
**Femoral diaphysis BMAT**								----------	rho=0.45 p=0.19
**Femoral metaphysis BMAT**									----------

Supplemental Table 4: Baseline correlations between body composition parameters and hormonal parameters (female subjects). Pearson correlation coefficients were calculated for normally distributed data and are represented by R. Spearman rank-order correlation coefficients were calculated for non-normally distributed data and are represented by rho.

Female subjects	**HOMA-IR**	**Leptin**	**Ghrelin**	**Adiponectin**	**FGF21**	**IGF-1**	**IGF-BP2**	**CTX**	**P1NP**	**GDF-15**	**IL-6**	**TNFα**	**CRP**	**G-CSF**
**BMI**	R= -0.32<br>p=0.36	R=0.12<br>p=0.73	**R= -0.70<br>p=0.02**	R= -0.52<br>p=0.12	R= -0.44<br>p=0.20	R=0.12<br>p=0.75	R= -0.29<br>p=0.42	**R= -0.68<br>p=0.03**	R= -0.44<br>p=0.20	R= -0.28<br>p=0.43	R=0.22<br>p=0.60	rho=0.39<br>p=0.26	rho= -0.62<br>p=0.06	R= -0.06<br>p=0.86
**SAT (MRI)**	R=0.28<br>p=0.44	R=0.61<br>p=0.06	R= -0.32<br>p=0.37	R=0.02<br>p=0.97	R= -0.41<br>p=0.23	R= -0.12<br>p=0.74	R= -0.17<br>p=0.63	R= -0.14<br>p=0.71	R=0.26<br>p=0.46	R=0.26<br>p=0.46	R=0.46<br>p=0.25	rho= -0.26<br>p=0.47	rho=0.44<br>p=0.20	R=0.18<br>p=0.61
**VAT (MRI)**	R=0.41<br>p=0.24	R=0.59<br>p=0.07	R= -0.52<br>p=0.12	R= -0.08<br>p=0.83	**R= -0.64<br>p=0.048**	R= -0.14<br>p=0.71	R=0.03<br>p=0.94	R= -0.27<br>p=0.44	R= -0.26<br>p=0.48	R=0.05<br>p=0.89	R=0.55<br>p=0.16	rho= -0.54<br>p=0.11	rho=0.40<br>p=0.25	R= -0.25<br>p=0.49
**Trunk fat (DXA)**	R=0.07<br>p=0.85	R=0.51<br>p=0.13	R= -0.40<br>p=0.25	R=0.10<br>p=0.77	R= -0.34<br>p=0.33	R= -0.30<br>p=0.40	R= -0.29<br>p=0.41	R= -0.21<br>p=0.57	R= -0.004<br>p=0.99	R=0.43<br>p=0.21	R=0.25<br>p=0.55	rho=0.21<br>p=0.56	rho=0.24<br>p=0.50	R=0.26<br>p=0.47
**Extremity fat (DXA)**	R= -0.25<br>p=0.48	R=0.02<br>p=0.96	R= -0.38<br>p=0.28	R= -0.37<br>p=0.30	R= -0.26<br>p=0.47	R= -0.03<br>p=0.93	R= -0.39<br>p=0.27	R= -0.03<br>p=0.93	R=0.37<br>p=0.29	R=0.18<br>p=0.62	R= -0.02<br>p=0.96	rho=0.43<br>p=0.21	rho= -0.22<br>p=0.54	R=0.44<br>p=0.20
**Lean mass (DXA)**	R=0.05<br>p=0.89	R= -0.17<br>p=0.64	R=0.30<br>p=0.41	R=0.19<br>p=0.59	R=0.46<br>p=0.18	R=0.20<br>p=0.59	R= -0.02<br>p=0.96	R= -0.05<br>p=0.90	R= -0.02<br>p=0.96	R=0.37<br>p=0.30	R=0.09<br>p=0.83	rho=0.62<br>p=0.05	rho=0.11<br>p=0.76	R=0.12<br>p=0.74
**L4 vertebra BMAT**	**R=0.67<br>p=0.03**	R=0.30<br>p=0.40	R= -0.46<br>p=0.18	R= -0.19<br>p=0.59	**R= -0.64<br>p=0.048**	R=0.17<br>p=0.64	R= -0.24<br>p=0.51	R= -0.22<br>p=0.54	R=0.09<br>p=0.81	R=0.22<br>p=0.54	R=0.56<br>p=0.15	rho= -0.01<br>p=0.99	rho=0.24<br>p=0.50	R= -0.01<br>p=0.97
**Femoral diaphysis BMAT**	R= 0.25<br>p=0.48	R= -0.46<br>p=0.18	R=0.40<br>p=0.25	R= -0.22<br>p=0.55	R=0.32<br>p=0.37	R=0.58<br>p=0.08	**R=0.71<br>p=0.02**	R=0.37<br>p=0.30	R=0.20<br>p=0.57	R= -0.16<br>p=0.65	R= -0.10<br>p=0.82	rho= -0.42<br>p=0.23	rho=0.04<br>p=0.91	R= -0.49<br>p=0.15
**Femoral metaphysis BMAT**	rho=0.39<br>p=0.26	rho=0.10<br>p=0.78	rho= -0.05<br>p=0.88	rho= -0.31<br>p=0.38	rho= -0.41<br>p=0.24	rho=0.39<br>p=0.27	rho=0.45<br>p=0.19	rho=0.07<br>p=0.85	rho=0.10<br>p=0.78	rho= -0.08<br>p=0.83	rho=0.21<br>p=0.61	rho= -0.58<br>p=0.08	rho=0.29<br>p=0.42	rho= -0.35<br>p=0.33

Supplemental Table 5: Baseline correlations between body composition parameters (male subjects). Pearson correlation coefficients were calculated for normally distributed data and are represented by R. Spearman rank-order correlation coefficients were calculated for non-normally distributed data and are represented by rho.

Male subjects	BMI	SAT	VAT	Trunk fat	Extremity fat	Lean mass	L4 BMAT	Femoral diaphysis BMAT	Femoral metaphysis BMAT
**BMI**	----------	**rho=0.73 p=0.005**	**rho=0.59 p=0.03**	**rho=0.75 p=0.003**	**rho=0.58 p=0.04**	rho=0.37 p=0.22	rho= -0.03 p=0.91	**rho= -0.57 p=0.04**	rho=0.25 p=0.43
**SAT**		----------	**R=0.81 p=0.0007**	**R=0.89 P<0.0001**	**R=0.69 p=0.009**	R= 0.09 p=0.77	R=0.20 p=0.50	R= -0.17 p=0.58	R=0.53 p=0.08
**VAT**			----------	**R=0.90 P<0.0001**	R=0.49 p=0.09	R= -0.05 p=0.88	R=0.10 p=0.76	R= -0.36 p=0.22	R=0.19 p=0.56
**Trunk fat**				----------	**R=0.69 p=0.009**	R= 0.03 p=0.93	R=0.34 p=0.26	R= -0.37 p=0.21	R=0.43 p=0.16
**Extremity fat**					----------	R= 0.44 p=0.13	R=0.17 p=0.58	R= -0.21 p=0.50	R=0.44 p=0.16
**Lean mass**						----------	R=0.03 p=0.91	R= 0.01 p=0.97	R=0.11 p=0.73
**L4 BMAT**							----------	R= 0.09 p=0.76	R=0.37 p=0.24
**Femoral diaphysis BMAT**								----------	R=0.21 p=0.52
**Femoral metaphysis BMAT**									----------

Supplemental Table 6: Baseline correlations between body composition parameters and hormonal parameters (male subjects). Pearson correlation coefficients were calculated for normally distributed data and are represented by R. Spearman rank-order correlation coefficients were calculated for non-normally distributed data and are represented by rho.

Male subjects	HOMA-IR	Leptin	Ghrelin	Adiponectin	FGF21	IGF-1	IGF-BP2	CTX	P1NP	GDF-15	IL-6	TNFα	CRP	G-CSF
**BMI**	rho=0.42<br>p=0.16	rho=0.38<br>p=0.20	rho= -0.38<br>p=0.19	rho= -0.42<br>p=0.16	rho= -0.19<br>p=0.56	rho= -0.33<br>p=0.27	**rho= -0.57**<br>**p=0.04**	rho= -0.35<br>p=0.24	rho=0.24<br>p=0.44	rho= -0.23<br>p=0.45	rho= -0.05<br>p=0.88	rho=0.18<br>p=0.55	rho=0.49<br>p=0.11	rho=0.42<br>p=0.15
**SAT (MRI)**	**rho=0.62**<br>**p=0.02**	rho=0.12<br>p=0.71	R= -0.15<br>p=0.61	R= -0.43<br>p=0.14	rho=0.01<br>p=0.97	**R= -0.56**<br>**p=0.045**	**R= -0.67**<br>**p=0.01**	R= -0.02<br>p=0.96	rho=0.25<br>p=0.42	R= -0.13<br>p=0.67	rho= -0.18<br>p=0.57	rho= -0.13<br>p=0.68	**rho=0.77**<br>**p=0.004**	rho=0.20<br>p=0.51
**VAT (MRI)**	rho=0.35<br>p=0.25	rho= -0.05<br>p=0.87	R= -0.23<br>p=0.46	R= -0.05<br>p=0.87	rho= -0.17<br>p=0.60	R= -0.44<br>p=0.13	R= -0.50<br>p=0.08	R=0.20<br>p=0.51	rho=0.37<br>p=0.22	R= -0.21<br>p=0.49	rho= -0.39<br>p=0.21	rho= -0.34<br>p=0.25	**rho=0.71**<br>**p=0.01**	rho=0.10<br>p=0.75
**Trunk fat (DXA)**	**rho=0.57**<br>**p=0.04**	rho=0.16<br>p=0.59	R= -0.26<br>p=0.40	R= -0.16<br>p=0.60	rho= -0.17<br>p=0.59	R= -0.44<br>p=0.13	**R= -0.61**<br>**p=0.03**	R=0.03<br>p=0.93	rho=0.27<br>p=0.37	R= -0.03<br>p=0.91	rho= -0.29<br>p=0.37	rho= -0.15<br>p=0.62	**rho=0.73**<br>**p=0.007**	rho=0.19<br>p=0.54
**Extremity fat (DXA)**	**rho=0.62**<br>**p=0.03**	rho=0.44<br>p=0.13	R=0.01<br>p=0.99	R= -0.24<br>p=0.43	rho= -0.34<br>p=0.29	R= -0.19<br>p=0.52	**R= -0.69**<br>**p=0.009**	R= -0.34<br>p=0.26	rho= -0.10<br>p=0.73	R=0.20<br>p=0.51	rho= -0.29<br>p=0.37	rho= -0.08<br>p=0.79	**rho=0.63**<br>**p=0.03**	rho=0.04<br>p=0.89
**Lean mass (DXA)**	rho=0.42<br>p=0.16	rho=0.38<br>p=0.19	R=0.07<br>p=0.81	R= -0.32<br>p=0.29	rho=0.22<br>p=0.50	R=0.32<br>p=0.29	R= -0.41<br>p=0.17	R= -0.43<br>p=0.14	rho= -0.37<br>p=0.21	R= -0.03<br>p=0.93	rho= -0.10<br>p=0.76	rho=0.35<br>p=0.25	rho=0.07<br>p=0.84	rho=0.47<br>p=0.10
**L4 vertebra BMAT**	rho=0.25<br>p=0.42	rho=0.08<br>p=0.79	R=0.04<br>p=0.90	R=0.09<br>p=0.76	rho= -0.25<br>p=0.43	R= -0.03<br>p=0.93	R= -0.02<br>p=0.95	R=0.30<br>p=0.31	rho=0.13<br>p=0.67	R=0.32<br>p=0.29	rho= -0.20<br>p=0.54	rho= -0.15<br>p=0.63	rho=0.06<br>p=0.86	rho= -0.20<br>p=0.52
**Femoral diaphysis BMAT**	rho= -0.20<br>p=0.51	rho= -0.46<br>p=0.11	**R=0.56**<br>**p=0.045**	R= -0.21<br>p=0.49	rho=0.57<br>p=0.05	R=0.46<br>p=0.11	R=0.02<br>p=0.95	R=0.22<br>p=0.48	rho= -0.30<br>p=0.32	R=0.29<br>p=0.33	rho=0.17<br>p=0.59	rho= -0.05<br>p=0.86	rho= -0.29<br>p=0.36	rho=0.07<br>p=0.82
**Femoral metaphysis BMAT**	rho=0.43<br>p=0.17	rho=0.08<br>p=0.81	R= -0.20<br>p=0.53	R= -0.56<br>p=0.06	rho=0.05<br>p=0.89	R= -0.15<br>p=0.64	R= -0.42<br>p=0.17	R= -0.46<br>p=0.13	rho= -0.31<br>p=0.33	R=0.27<br>p=0.39	rho=0.39<br>p=0.23	rho=0.25<br>p=0.43	rho=0.29<br>p=0.39	rho=0.12<br>p=0.71

Supplemental Table 7: Univariate correlations between % changes in body composition parameters and % change in hormonal parameters during high-calorie visit. Pearson correlation coefficients were calculated for normally distributed data and are represented by R. Spearman rank-order correlation coefficients were calculated for non-normally distributed data and are represented by rho.

	**% change in HOMA-IR**	**% change in leptin**	**% change in ghrelin**	**% change in adiponectin**	**% change in FGF21**	**% change in IGF-1**	**% change in IGF-BP2**	**% change in CTX**	**% change in P1NP**	**% change in GDF-15**	**% change in IL-6**	**% change in TNFα**	**% change in CRP**	**% change in G-CSF**
**% change in weight**	rho=0.12<br>*p*=0.59	rho=0.16<br>*p*=0.45	R=-0.10<br>*p*=0.66	R=-0.24<br>*p*=0.27	R=-0.13<br>*p*=0.56	R=0.30<br>*p*=0.16	rho= -0.20<br>*p*=0.37	rho= -0.32<br>*p*=0.13	rho=-0.18<br>*p*=0.42	rho=0.14<br>*p*=0.53	rho=-0.07<br>*p*=0.76	R=0.33<br>*p*=0.12	rho=0.37<br>*p*=0.09	rho=0.27<br>*p*=0.22
**% change in SAT (MRI)**	rho=0.37<br>*p*=0.08	rho=0.30<br>*p*=0.17	R=-0.01<br>*p*=0.97	R=0.33<br>*p*=0.13	R=0.30<br>*p*=0.17	R=0.05<br>*p*=0.82	rho= -0.31<br>*p*=0.16	rho= -0.11<br>*p*=0.62	rho=0.37<br>*p*=0.09	rho=-0.13<br>*p*=0.57	**rho=-0.47**<br>***p*=0.04**	R=-0.35<br>*p*=0.10	**rho=0.73**<br>***p*=0.0001**	rho=0.21<br>*p*=0.34
**% change in VAT (MRI)**	rho=0.20<br>*p*=0.36	rho=0.01<br>*p*=0.96	R=-0.20<br>*p*=0.36	R=0.10<br>*p*=0.65	R=-0.17<br>*p*=0.45	R=0.23<br>*p*=0.29	rho=0.06<br>*p*=0.79	rho= -0.25<br>*p*=0.26	rho=-0.22<br>*p*=0.32	rho=0.27<br>*p*=0.22	rho=0.37<br>*p*=0.11	R=0.22<br>*p*=0.32	rho=0.04<br>*p*=0.87	rho=-0.04<br>*p*=0.85
**% change in trunk fat (DXA)**	rho=0.13<br>*p*=0.57	rho= -0.07<br>*p*=0.74	R=0.11<br>*p*=0.62	R=0.07<br>*p*=0.76	R=0.02<br>*p*=0.94	R=0.02<br>*p*=0.93	rho= -0.17<br>*p*=0.45	rho=0.13<br>*p*=0.54	rho=-0.26<br>*p*=0.23	rho=0.03<br>*p*=0.88	rho=-0.01<br>*p*=0.95	R=-0.29<br>*p*=0.17	**rho=0.48**<br>***p*=0.03**	rho=0.19<br>*p*=0.38
**% change in extremity fat (DXA)**	rho= -0.02<br>*p*=0.93	rho=0.16<br>*p*=0.48	R=0.18<br>*p*=0.42	R=-0.02<br>*p*=0.92	R=0.23<br>*p*=0.30	R=0.16<br>*p*=0.47	rho=0.08<br>*p*=0.72	rho=0.01<br>*p*=0.97	rho=-0.20<br>*p*=0.36	rho=-0.15<br>*p*=0.51	rho=-0.03<br>*p*=0.91	R=-0.35<br>*p*=0.11	**rho=0.45**<br>***p*=0.03**	rho=0.04<br>*p*=0.86
**% change in lean mass (DXA)**	rho=0.18<br>*p*=0.41	rho=0.13<br>*p*=0.55	R=-0.34<br>*p*=0.11	R=-0.10<br>*p*=0.66	R=-0.18<br>*p*=0.43	R=0.29<br>*p*=0.19	rho= -0.20<br>*p*=0.36	rho= -0.37<br>*p*=0.09	rho=-0.10<br>*p*=0.63	rho=0.15<br>*p*=0.48	rho=-0.19<br>*p*=0.44	R=0.32<br>*p*=0.14	rho=0.18<br>*p*=0.43	rho=0.31<br>*p*=0.15
**% change in L4 vertebra BMAT**	**rho=0.55**<br>***p*=0.007**	rho=0.14<br>*p*=0.52	**rho=-0.62**<br>**p=0.002**	rho=0.20<br>*p*=0.35	rho=-0.40<br>*p*=0.07	rho=0.01<br>*p*=0.96	**rho= -0.44**<br>***p*=0.04**	rho=0.06<br>*p*=0.78	rho=-0.004<br>*p*=0.99	rho=0.11<br>*p*=0.62	rho=-0.26<br>*p*=0.28	rho=-0.13<br>*p*=0.54	rho=0.30<br>*p*=0.18	rho=0.03<br>*p*=0.88
**% change in femoral diaphysis BMAT**	rho=0.24<br>*p*=0.29	rho=0.06<br>*p*=0.78	R=-0.34<br>*p*=0.12	R=-0.05<br>*p*=0.82	R=-0.10<br>*p*=0.66	R=-0.09<br>*p*=0.70	rho= -0.19<br>*p*=0.39	rho= -0.23<br>*p*=0.29	rho=-0.22<br>*p*=0.32	rho=0.22<br>*p*=0.33	rho=0.10<br>*p*=0.68	R=-0.11<br>*p*=0.61	rho=0.03<br>*p*=0.88	rho=0.31<br>*p*=0.17
**% change in femoral metaphysis BMAT**	rho=0.12<br>*p*=0.59	rho= -0.03<br>*p*=0.91	R=-0.13<br>*p*=0.58	R=-0.19<br>*p*=0.40	R=0.03<br>*p*=0.89	R=0.02<br>*p*=0.92	rho= -0.20<br>*p*=0.38	rho= -0.39<br>*p*=0.08	rho=0.001<br>*p*=0.996	rho=-0.25<br>*p*=0.27	rho=-0.19<br>*p*=0.47	R=-0.26<br>*p*=0.25	rho=0.23<br>*p*=0.33	rho=0.30<br>*p*=0.18

Supplemental Table 8: Univariate correlations between % changes in body composition parameters and % change in hormonal parameters during fasting visit. Pearson correlation coefficients were calculated for normally distributed data and are represented by R. Spearman rank-order correlation coefficients were calculated for non-normally distributed data and are represented by rho.

	**% change in HOMA-IR**	**% change in leptin**	**% change in ghrelin**	**% change in adiponectin**	**% change in FGF21**	**% change in IGF-1**	**% change in IGF-BP2**	**% change in CTX**	**% change in P1NP**	**% change in GDF-15**	**% change in IL-6**	**% change in TNFα**	**% change in CRP**	**% change in G-CSF**
**% change in weight**	rho=-0.03<br>*p*=0.90	**R=-0.43<br>*p*=0.04**	R=0.14<br>*p*=0.55	rho=0.12<br>*p*=0.58	rho=0.23<br>*p*=0.29	rho=0.19<br>*p*=0.39	R=0.17<br>*p*=0.44	rho= -0.05<br>*p*=0.83	R=-0.05<br>*p*=0.82	R=-0.12<br>*p*=0.59	**rho=-0.50<br>*p*=0.02**	R=-0.09<br>*p*=0.69	rho=-0.23<br>*p*=0.31	R=-0.17<br>*p*=0.44
**% change in SAT (MRI)**	rho=-0.02<br>*p*=0.95	rho=0.002<br>*p*=0.99	rho=-0.39<br>*p*=0.09	rho=0.14<br>*p*=0.52	rho=-0.01<br>*p*=0.95	rho=0.31<br>*p*=0.15	rho=-0.06<br>*p*=0.79	rho= 0.30<br>*p*=0.17	rho=-0.29<br>*p*=0.19	rho=-0.10<br>*p*=0.66	**rho=-0.42<br>*p*=0.045**	**rho=-0.61<br>*p*=0.002**	rho=0.10<br>*p*=0.66	rho=-0.31<br>*p*=0.15
**% change in VAT (MRI)**	rho=0.38<br>*p*=0.10	R=0.14<br>*p*=0.53	R=-0.31<br>*p*=0.18	rho=-0.34<br>*p*=0.11	rho=-0.34<br>*p*=0.11	rho=0.37<br>*p*=0.08	R=-0.04<br>*p*=0.87	rho= 0.14<br>*p*=0.53	R=-0.34<br>*p*=0.11	R=0.15<br>*p*=0.49	rho=0.15<br>*p*=0.51	R=-0.38<br>*p*=0.07	rho=0.004<br>*p*=0.99	R=0.08<br>*p*=0.73
**% change in trunk fat (DXA)**	rho=-0.12<br>*p*=0.60	R=-0.004<br>*p*=0.99	R=-0.18<br>*p*=0.44	rho=0.09<br>*p*=0.69	rho=0.28<br>*p*=0.19	rho=-0.09<br>*p*=0.67	R=-0.31<br>*p*=0.15	**rho=0.56<br>*p*=0.005**	R=-0.07<br>*p*=0.74	R=0.28<br>*p*=0.19	rho=-0.07<br>*p*=0.76	R=-0.25<br>*p*=0.25	**rho=0.49<br>*p*=0.02**	R=-0.05<br>*p*=0.84
**% change in extremity fat (DXA)**	rho= -0.14<br>*p*=0.56	R=-0.09<br>*p*=0.68	R=-0.08<br>*p*=0.74	**rho=0.48<br>*p*=0.02**	rho=0.15<br>*p*=0.49	rho=-0.05<br>*p*=0.81	**R=-0.49<br>*p*=0.02**	rho=0.33<br>*p*=0.12	R=0.10<br>*p*=0.63	R=0.05<br>*p*=0.83	rho=-0.23<br>*p*=0.29	R=-0.16<br>*p*=0.46	rho=0.18<br>*p*=0.43	R=-0.28<br>*p*=0.21
**% change in lean mass (DXA)**	rho=0.20<br>*p*=0.38	R=-0.34<br>*p*=0.12	R=0.05<br>*p*=0.83	rho=-0.04<br>*p*=0.85	rho=0.05<br>*p*=0.81	rho=0.20<br>*p*=0.36	R=0.30<br>*p*=0.16	rho= -0.08<br>*p*=0.72	R=-0.13<br>*p*=0.55	R=-0.22<br>*p*=0.32	rho=0.17<br>*p*=0.44	R=-0.001<br>*p*=0.996	rho=-0.31<br>*p*=0.16	R=-0.04<br>*p*=0.86
**% change in L4 vertebra BMAT**	rho=-0.01<br>*p*=0.95	R=-0.21<br>*p*=0.35	R=0.06<br>*p*=0.81	rho=-0.004<br>*p*=0.99	rho=-0.28<br>*p*=0.19	rho=0.14<br>*p*=0.53	R=0.02<br>*p*=0.92	rho=0.10<br>*p*=0.65	R=0.19<br>*p*=0.39	R=0.06<br>*p*=0.78	rho=0.31<br>*p*=0.15	R=-0.12<br>*p*=0.57	rho=-0.11<br>*p*=0.63	R=0.40<br>*p*=0.07
**% change in femoral diaphysis BMAT**	rho=0.32<br>*p*=0.17	R=0.13<br>*p*=0.56	R=-0.07<br>*p*=0.77	rho=-0.02<br>*p*=0.94	rho=-0.16<br>*p*=0.46	rho=0.26<br>*p*=0.23	R=-0.14<br>*p*=0.53	rho=-0.004<br>*p*=0.99	R=0.31<br>*p*=0.15	R=-0.17<br>*p*=0.44	rho=0.02<br>*p*=0.94	R=0.002<br>*p*=0.99	rho=-0.11<br>*p*=0.63	R=0.15<br>*p*=0.51
**% change in femoral metaphysis BMAT**	rho=0.14<br>*p*=0.57	R=0.09<br>*p*=0.68	R=0.01<br>*p*=0.98	rho=0.11<br>*p*=0.61	rho=0.01<br>*p*=0.96	rho=0.24<br>*p*=0.27	R=-0.05<br>*p*=0.81	rho= -0.11<br>*p*=0.63	R=0.19<br>*p*=0.39	R=-0.29<br>*p*=0.18	rho=-0.12<br>*p*=0.59	R=-0.06<br>*p*=0.80	rho=0.30<br>*p*=0.18	R=-0.07<br>*p*=0.76

Supplemental Table 9: Univariate correlations between % changes in body composition parameters and % change in hormonal parameters during two-week stabilization period between high-calorie and fasting visits. Pearson correlation coefficients were calculated for normally distributed data and are represented by R. Spearman rank-order correlation coefficients were calculated for non-normally distributed data and are represented by rho.

	**% change in HOMA-IR**	**% change in leptin**	**% change in ghrelin**	**% change in adiponectin**	**% change in FGF21**	**% change in IGF-1**	**% change in IGF-BP2**	**% change in CTX**	**% change in P1NP**	**% change in GDF-15**	**% change in IL-6**	**% change in TNFα**	**% change in CRP**	**% change in G-CSF**
**% change in weight**	rho=0.18 *p*=0.42	R=0.17 *p*=0.44	rho=0.001 *p*=0.996	R=0.33 *p*=0.13	rho=0.25 *p*=0.24	R=0.10 *p*=0.64	rho= -0.28 *p*=0.20	rho= -0.34 *p*=0.11	R=0.22 *p*=0.32	rho=0.30 *p*=0.17	rho=0.02 *p*=0.92	R=-0.13 *p*=0.55	rho=0.31 *p*=0.16	rho=-0.12 *p*=0.59
**% change in SAT (MRI)**	rho=0.003 *p*=0.99	R=0.18 *p*=0.40	rho=-0.15 *p*=0.51	R=0.26 *p*=0.22	rho=-0.09 *p*=0.67	R=-0.04 *p*=0.85	rho= 0.10 *p*=0.66	rho= -0.24 *p*=0.28	**R=0.47 *p*=0.02**	rho=0.02 *p*=0.94	rho=-0.18 *p*=0.44	R=-0.27 *p*=0.21	rho=-0.03 *p*=0.88	rho=-0.13 *p*=0.54
**% change in VAT (MRI)**	rho=0.11 *p*=0.63	rho=0.24 *p*=0.26	rho=-0.09 *p*=0.69	rho=0.31 *p*=0.15	rho=-0.08 *p*=0.71	rho=-0.25 *p*=0.25	rho=-0.30 *p*=0.16	rho= 0.10 *p*=0.64	rho=0.25 *p*=0.25	rho=0.39 *p*=0.07	**rho=0.44 *p*=0.04**	**rho=0.46 *p*=0.03**	rho=0.17 *p*=0.44	rho=0.05 *p*=0.82
**% change in trunk fat (DXA)**	rho=0.11 *p*=0.63	R=0.03 *p*=0.91	rho=0.02 *p*=0.94	R=0.34 *p*=0.11	rho=0.20 *p*=0.36	R=-0.05 *p*=0.81	**rho=0.47 *p*=0.02**	rho=-0.30 *p*=0.16	R=0.41 *p*=0.053	rho=0.17 *p*=0.44	rho=0.09 *p*=0.71	R=-0.12 *p*=0.59	rho=0.34 *p*=0.12	**rho=-0.43 *p*=0.04**
**% change in extremity fat (DXA)**	rho= 0.12 *p*=0.60	rho=0.30 *p*=0.16	rho=0.20 *p*=0.35	rho=0.05 *p*=0.82	rho=0.08 *p*=0.72	rho=0.16 *p*=0.45	rho=0.07 *p*=0.75	rho=-0.28 *p*=0.20	rho=0.33 *p*=0.12	rho=0.05 *p*=0.84	rho=0.15 *p*=0.52	rho=-0.26 *p*=0.23	rho=0.06 *p*=0.77	rho=0.03 *p*=0.90
**% change in lean mass (DXA)**	rho=0.02 *p*=0.91	R=0.09 *p*=0.68	rho=-0.14 *p*=0.52	R=0.19 *p*=0.38	rho=0.17 *p*=0.42	R=0.13 *p*=0.56	rho= -0.22 *p*=0.31	rho= -0.15 *p*=0.49	R=-0.13 *p*=0.54	rho=0.35 *p*=0.10	rho=-0.01 *p*=0.96	R=0.11 *p*=0.63	rho=0.08 *p*=0.74	rho=-0.16 *p*=0.46
**% change in L4 vertebra BMAT**	rho=0.30 *p*=0.17	R=-0.08 *p*=0.71	rho=-0.40 *p*=0.055	R=0.25 *p*=0.25	rho=0.02 *p*=0.92	R=-0.10 *p*=0.66	rho= 0.05 *p*=0.82	rho=-0.07 *p*=0.75	R=0.08 *p*=0.72	rho=0.09 *p*=0.68	rho=-0.22 *p*=0.35	R=-0.25 *p*=0.24	rho=0.11 *p*=0.63	rho=-0.25 *p*=0.25
**% change in femoral diaphysis BMAT**	rho=-0.15 *p*=0.51	R=0.30 *p*=0.17	rho=-0.07 *p*=0.77	R=0.17 *p*=0.44	**rho=0.48 *p*=0.03**	R=0.12 *p*=0.58	rho= 0.14 *p*=0.53	rho= 0.16 *p*=0.49	R=-0.02 *p*=0.94	rho=-0.09 *p*=0.71	rho=0.10 *p*=0.69	R=0.14 *p*=0.52	rho=0.05 *p*=0.83	**rho=-0.52 *p*=0.01**
**% change in femoral metaphysis BMAT**	rho=-0.01 *p*=0.98	R=0.31 *p*=0.16	rho=0.09 *p*=0.68	R=0.27 *p*=0.22	rho=0.31 *p*=0.16	R=0.13 *p*=0.57	rho= -0.10 *p*=0.69	rho= 0.02 *p*=0.92	R=0.09 *p*=0.68	rho=-0.34 *p*=0.12	rho=-0.29 *p*=0.21	R=-0.14 *p*=0.52	rho=0.17 *p*=0.46	rho=-0.39 *p*=0.07

Supplemental Table 10: Semi-quantitative real-time RT-PCR: total RNA from human mature adipocytes was isolated. Mature adipocytes were analyzed by RT-PCR using the primer sets outlined below. Results from each mRNA were normalized relative to the expression of GAPDH as a control.

**GENE**	**FORWARD**	**REVERSE**
GAPDH	GGCATGGACCTGTGGTCATGAG	TGCACCACCAACTGCTTAGC
***RETN* (resistin)**	CTCAGGGCTGCACACGACAG	CCGAGGCTTCGCCGTCAC
***PLXND1* (PlexinD1)**	TATCTGTCAGGCAGGGGTTC	CCTGGGTCACCTCTGTGTTT
SEMA3E	AAGGTCAGATTCCATCACTGTGACAT	AGCAAAGTACTGTTGTTCTCTATGC
***TNF* (TNFα)**	ACAAGCCTGTAGCCCATGTT	AAAGTAGACCTGCCCAGACT

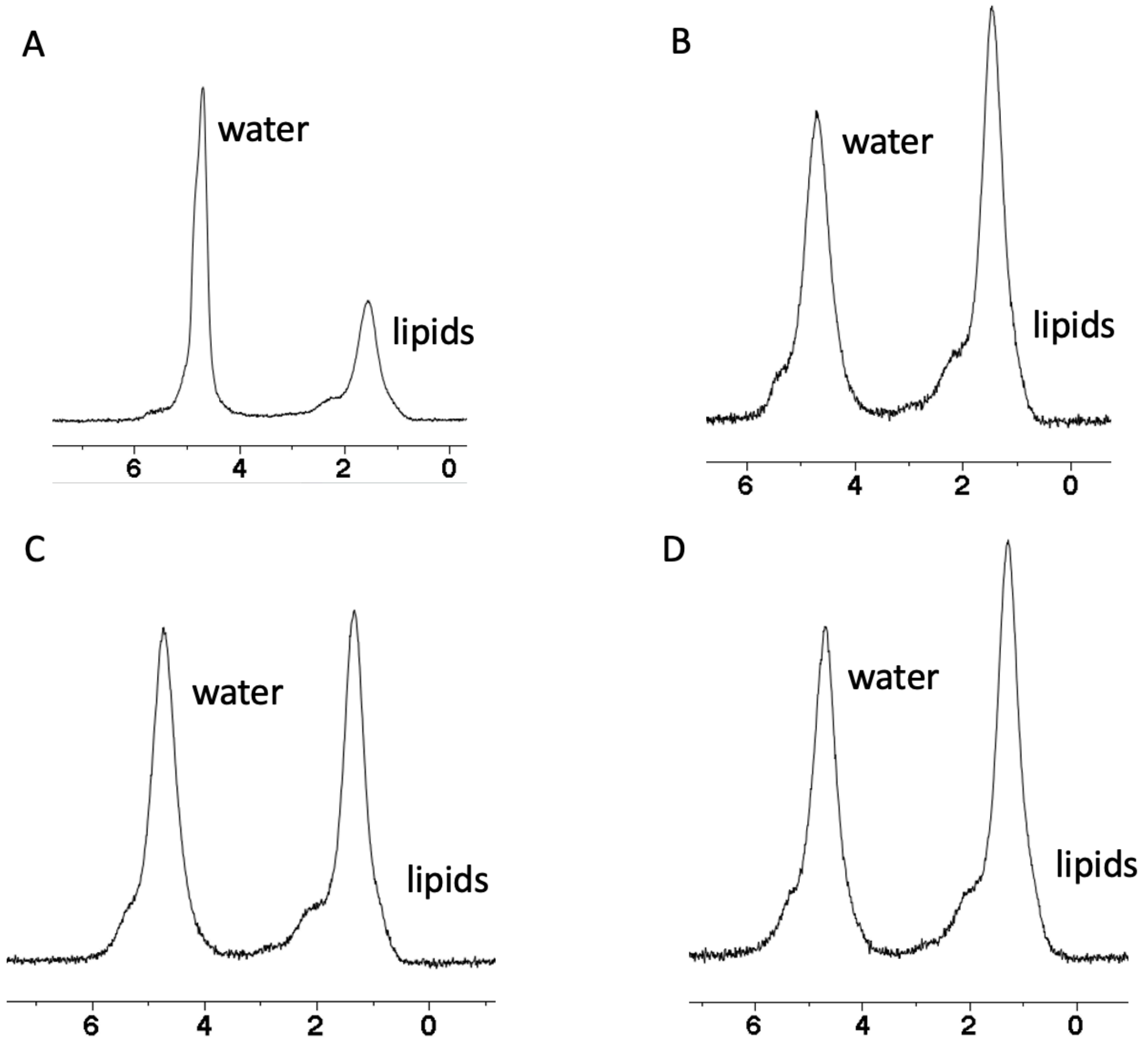

Supplemental Figure 1: Proton magnetic resonance spectroscopy (^{1}H-MRS) of the L4 vertebra for assessment of bone marrow adipose tissue (BMAT) content expressed in lipid-to-water ratio (LWR). BMAT content in a 31-year-old man prior to (Panel A) and 10 days after high-calorie feeding (Panel B) showing an increase in BMAT content (0.57 LWR pre vs. 1.30 LWR post). BMAT content decreased following the two-week stabilization period (Panel C) and increased after 10 days of fasting (Panel D) (1.10 LWR pre vs. 1.42 LWR post). For purposes of visual comparison, the amplitudes of unsuppressed water are scaled identically.
