# Supplementary material for: The dynamics of human bone marrow adipose tissue in response to feeding and fasting
Source: JCI Insight. 2021 Jun 22;6(12):e138636. doi: 10.1172/jci.insight.138636 (PMC8262500; doi:10.1172/jci.insight.138636)
Supplement: Trial reporting checklists [file jciinsight-6-138636-s106.pdf]

Non-clinical trial human subjects research. Primary outcome (change in bone marrow adipose tissue) is not a clinically relevant outcome at the present time.
